# Metabolic alterations upon SARS-CoV-2 infection and potential therapeutic targets against coronavirus infection

**DOI:** 10.1038/s41392-023-01510-8

**Published:** 2023-06-07

**Authors:** Peiran Chen, Mandi Wu, Yaqing He, Binghua Jiang, Ming-Liang He

**Affiliations:** 1 Department of Biomedical Sciences, City University of Hong Kong, HKSAR, Hong Kong, China; 2 Shenzhen Center for Disease Control and Prevention, Shenzhen, 518055 Guangdong China; 3 Cell Signaling and Proteomic Center, Academy of Medical Sciences, Zhengzhou University, Zhengzhou, Henan China

**Keywords:** Microbiology, Infectious diseases

## Abstract

The coronavirus disease 2019 (COVID-19) caused by coronavirus SARS-CoV-2 infection has become a global pandemic due to the high viral transmissibility and pathogenesis, bringing enormous burden to our society. Most patients infected by SARS-CoV-2 are asymptomatic or have mild symptoms. Although only a small proportion of patients progressed to severe COVID-19 with symptoms including acute respiratory distress syndrome (ARDS), disseminated coagulopathy, and cardiovascular disorders, severe COVID-19 is accompanied by high mortality rates with near 7 million deaths. Nowadays, effective therapeutic patterns for severe COVID-19 are still lacking. It has been extensively reported that host metabolism plays essential roles in various physiological processes during virus infection. Many viruses manipulate host metabolism to avoid immunity, facilitate their own replication, or to initiate pathological response. Targeting the interaction between SARS-CoV-2 and host metabolism holds promise for developing therapeutic strategies. In this review, we summarize and discuss recent studies dedicated to uncovering the role of host metabolism during the life cycle of SARS-CoV-2 in aspects of entry, replication, assembly, and pathogenesis with an emphasis on glucose metabolism and lipid metabolism. Microbiota and long COVID-19 are also discussed. Ultimately, we recapitulate metabolism-modulating drugs repurposed for COVID-19 including statins, ASM inhibitors, NSAIDs, Montelukast, omega-3 fatty acids, 2-DG, and metformin.

## Introduction

In the 21st century, coronavirus infections have become major global challenges not only to public health, but also to government managements. Just in the last two decades, we have experienced three pandemic outbreaks caused by ß-coronavirus infections. Coronaviruses are enveloped viruses containing an ~30 kb positive-sense single-stranded RNA [(+) ssRNA] genome.^1^ Coronaviruses transmit among different species, including humans, livestock, and wild animals. An epidemic caused by SARS-CoV outbroke in China in 2002-2003, culminating in 774 reported casualties. Middle East respiratory syndrome coronavirus (MERS-CoV) accounts for another global outbreak in 2020 with over 800 associated deaths.^2^ Since December 2019, coronavirus disease 2019 (COVID-19) has caused a global pandemic with symptoms of pneumonia, nausea, fever, and respiratory system impairment.^3,4^ COVID-19 is caused by a novel pathogen, namely severe acute respiratory syndrome-coronavirus 2 (SARS-CoV-2). Since 2019, COVID-19 has brought unprecedented casualties and socioeconomic burden.^4,5^

Most COVID-19 cases are asymptomatic or mild. However, some patients have courses characterized by a generalized inflammatory state causing tissue injury in multiple organs and ARDS with a global mortality rate of 3.4%.^6^ Patients with hypertension, diabetes, and cardiovascular diseases have a higher risk of mortality.^7,8^ Hitherto, due to a paucity of validated modalities or vaccines, COVID-19 remains a horrendous threat worldwide. Despite enormous scientific efforts that have been dedicated to SARS-CoV-2, the deep layers of SARS-CoV-2 biology and pathogenesis are not yet well understood.

The envelope (E), membrane (M), and spike (S) proteins together comprise the outer shield of SARS-CoV-2; while the core of SARS-COV-2 consists of viral genomic RNA condensed by nucleocapsid (N) protein. The viral genome RNA encodes non-structural proteins (NSPs), structural proteins (E, M, S, and N) and accessory proteins. NSPs are functional in viral RNA replication, protein synthesis, and regulating intracellular signaling pathways.^9,10^ NSPs also play crucial roles in attenuating host innate immunity to facilitate the escape from host defense and initiate inflammatory response.^11^ S gives rise to the corona shape of the surface and mediates host receptor recognition and viral entry. SARS-CoV-2 specifically recognizes and attaches human angiotensin-converting enzyme 2 (ACE2) for entry via S protein.^12,13^ E mediates virus assembly, membrane scission, and budding, playing a pivotal role in virus replication and intercellular transmission.^14^ M is the most abundant protein in the envelope that directs the assembly process through interaction with the other structural proteins.^15^ N directly binds with viral RNA, serving as capsulation to protect viral RNA from cytoplasmic immune surveillance and to mediate nucleoprotein complex assembly.^16–18^

**Fig. 1:**
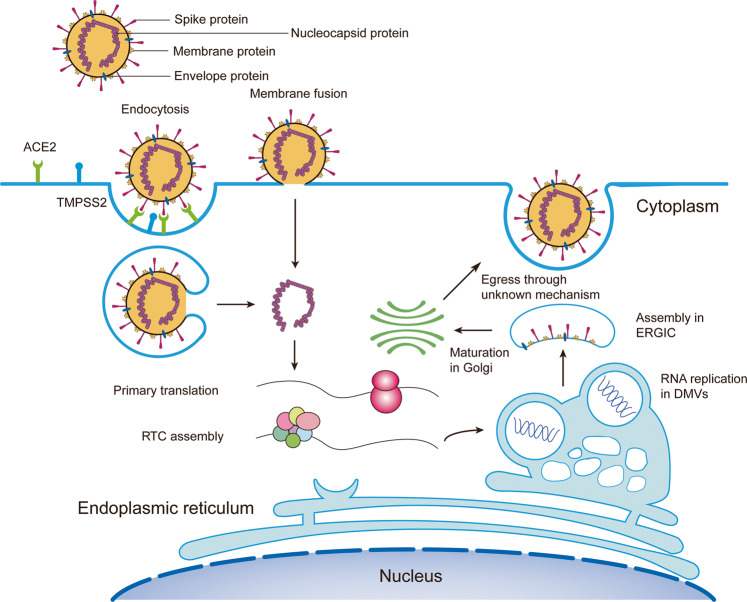
The life cycle of SARS-CoV-2. The life cycle of SARS-CoV-2 includes the following stages: receptor recognition via S protein; viral entry through the endocytosis pathway or the membrane fusion pathway; replicative transcription complex formation; viral RNA replication in DMVs; virion assembly in ERGIC; virion maturation in Golgi; egress through an unknown pathway

The life cycle of coronaviruses generally includes the following stages (as shown in Fig. 1): host cell receptor specific engagement with S protein^19^; viral uptake by endocytosis or membrane fusion^13^; uncoating and viral RNA synthesis in replication organelles^20,21^; progeny virions assembly to mature virions; ultimate release by unknown mechanism.^3^ Following viral entry by membrane fusion or endocytosis, the release and uncoating of viral genomic RNA, together with NSPs, subject to translation and formation of replication and translation complex (RTC). In the replication stage, viral genomic RNA is coated by double-membrane vesicles (DMVs), which are derived from the endoplasmic reticulum (ER).^22^ DMVs provide a microenvironment to protect the viral genome and to facilitate viral RNA synthesis.^23,24^ DMVs have a pore to connect with the cytoplasm for material exchange while shielding viral RNA from intracellular immune surveillance.^25^ In the assembly stage, newly produced viral RNA and NSPs are subsequently incorporated into virions at the cytoplasmic side of the ER-to-Golgi intermediate compartment (ERGIC).^26,27^ After being transported to Golgi, coronaviruses are further modified and assembled, giving rise to the morphology of mature virions. Ultimately, virions egress from the host cell by exocytosis. As for the mechanism for viral egress, debates still exist. It was initially postulated that SARS-CoV-2, similar to other enveloped viruses, hijacks the biosynthetic secretory pathway for its exit. However, recent research discovers that coronaviruses including SARS-CoV-2 use lysosome for egress instead of hijacking the biosynthetic secretory pathway.^28^ Until now, the egress mechanism of coronaviruses has not been determined yet.^27,29^

Lipid metabolism is indispensable in providing energy and material, maintaining homeostasis, regulating immune response, and relaying signals. Many viruses were reported to rewire host cellular lipid metabolism to create replication compartments.^30–34^ Lipid droplet (LD), a cellular organelle for lipid storage, was reported to be closely associated with cellular antiviral innate immunity.^35,36^ Lipid accumulation was observed in the lungs of COVID-19 patients and cells infected by SARS-CoV-2, colocalizing with N protein.^37,38^ Significant lipid pattern alterations were demonstrated by Lipidomic analyses of plasma from COVID-19 patients, and the lipid pattern was shown to correlate with disease severity and progression.^39,40^

Glucose metabolism is another network that regulates a series of physiological alterations. Aside from providing energy and material for cells, glucose metabolism is also associated with virus infection, immunity, tumorigenesis, homeostasis, and so forth.^41–47^ More importantly, it is supported by clinical evidence that diabetes mellitus, which is characterized by an impaired capability to control blood glucose, is a major risk factor predisposing to severe COVID-19.^7,48,49^ A retrospective study shows that well-controlled blood glucose is associated with markedly lower mortality rates. On the contrary, patients with type 2 diabetes (T2D) or dysregulated blood glucose have poor outcomes.^50^ Analysis of single-cell RNA-sequencing data in bronchoalveolar lavage fluid immune cells reveals that enhanced glycolysis is the most important metabolic feature of all immune cells in COVID-19 patients.^51^ The relationship between dysregulated blood glucose profile and COVID-19 is bidirectional. Infection by SARS-CoV-2 could also worsen the condition of type 2 diabetes patients.^52,53^

Considering the huge socioeconomic damage caused by coronaviruses, it is an urgent need to develop effective therapeutic interventions for the next outbreak. A more profound comprehension of host metabolic alterations and their association with coronaviruses will prompt us to understand more about COVID-19 pathology and promote antiviral drug development. This review aims to generalize and discuss current findings about the role of host metabolism in SARS-CoV-2 infection.

### Virus entry

Apart from ACE2, other host receptors including lectins, DC-SIGN, L-SIGN, and AXL, also serve as alternative entry sites of SARS-CoV-2.^54–56^ S protein trimers recognize and bind to cell receptor ACE2 with its S1 domain. Then S protein is cleaved by host transmembrane serine protease TMPRSS2 at the S2’ site.^57^ The anchored S2 domain is activated to trigger viral and host lipids bilayer fusion, releasing the viral ribonucleoprotein complex into the cell.^58–60^ In addition to the membrane fusion pathway, endocytosis is also hijacked for SARS-CoV-2 entry.^61,62^ The endocytosis pathway mediates the trafficking of SARS-CoV-2 to the late endosome/lysosome, in which proteases (such as cathepsin L) prime S protein to initiate membrane fusion.^63,64^

Increasing evidence supports the critical role of *lipid rafts* in SARS-CoV-2 infection. Lipid rafts are located at cell membranes, they are microdomains enriched in lipid molecules including cholesterol and sphingolipids. Lipid rafts are involved in a variety of physiological processes.^65^ Lipid rafts are also proposed as important for the entry of other coronaviruses by providing a platform for entry receptors. Lipid rafts are suggested to be a promising therapeutic target.^66–68^ From a mechanistic perspective, lipid rafts provide platforms for membrane receptors involved in viral entry of SARS-CoV-2.^69^ SARS-CoV-2 entry can be reduced by disturbing lipid rafts. ACE2 colocalizes with well-established raft proteins caveolin-1, flotillin-2, and ganglioside GM1 in Vero E6 cells.^70,71^ However, either endogenous ACE2 in Vero E6 cells or the transiently expressed ACE2 in CHO cells is not enriched in lipid rafts.^72,73^ The controversial results might be caused by different experimental methods. How and which receptors are recruited to the lipid rafts requires further investigation.

Changes in cholesterol levels disrupt *lipid rafts* and the receptors attached.^74^ SARS-CoV-2 spike-bearing pseudovirus infection is associated with cholesterol-rich lipid rafts.^75^ Accordingly, Cholesterol-25-hydroxylase, an interferon-stimulated gene (ISG) that triggers cholesterol trafficking from the plasma membrane to ER, inhibits SARS-CoV-2 entry by depriving accessible cholesterol at the plasma membrane. More importantly, the entry inhibition can be restored by replenishing soluble cholesterol to the cells.^76^ Furthermore, lipid rafts are also proposed to promote viral entry through the endocytosis pathway. SARS-CoV-2 internalization is mediated through a lipid raft-dependent endocytic pathway, but which endocytosis pathway is practically responsible for SARS-CoV-2 entry entails further investigations.^75^ In some enveloped viruses, infection causes fusogenic viral protein displayed on the cell membrane, which allows adjacent cells to fuse and form multinucleated syncytia.^77,78^ Syncytia formation is also observed in SARS-CoV-2 infected cells or lungs of deceased patients.^79,80^ Syncytia formation indicates that SARS-CoV-2 has an ability of cell-to-cell transmission, allowing the virus to avoid contact with antibody.^81^ Of note, it is evident that cell-to-cell transmission through the formation of channels or syncytia requires intact lipid rafts.^82^ In addition, S protein-mediated membrane fusion and syncytia formation requires cholesterol involvement.^80^ Hence, it is reasonable to deduce that lipid rafts are also involved in syncytia formation during SARS-CoV-2 infection.

Intriguingly, S protein can directly interact with *cholesterol*. A study identified putative cholesterol recognition amino acid consensus motifs in SARS-CoV-2 S protein. Antibodies blocking the cholesterol-binding site of S protein significantly curbed viral entry.^83^ The interaction between S protein and high-density lipoprotein (HDL) has been interrogated. SR-B1 is an HDL receptor located on the cell membrane that drives the cellular uptake of cholesteryl esters and other lipid components of HDL.^84^ S protein directly binds with SR-B1-bound HDL and captures lipid materials from HDL. Genetic depletion of SR-B1 curbs SARS-CoV-2 pseudovirus entry.^83^ Another study also demonstrated that S protein can remove lipid components from HDL. Co-culture of HDL with S protein altered the function of HDL to exchange lipids from model cellular membranes.^85^

Although *cholesterol* on cell membrane facilitates viral entry, the role of intracellular cholesterol in SARS-CoV-2 infection is more complicated. Two independent genetical screens by CRISPR libraries identified genes in cholesterol metabolism as essential for SARS-CoV-2 infection, including sterol-regulatory element-binding protein (SREBP-2), SREBP cleavage activating protein (SCAP), low-density lipoprotein receptor (LDLR), and Membrane-Bound Transcription Factor Peptidase, site 1 and 2 (MBTPS1 and MBTPS2). Treatment of amlodipine, a calcium-channel antagonist that increases intracellular cholesterol levels, significantly inhibited SARS-CoV-2 infection.^86,87^ In addition to facilitating viral entry, it is likely that cholesterol metabolism also affects SARS-CoV-2 infection in other stages of its life cycle. Future studies on the interplay between SARS-CoV-2 and cholesterol metabolism are warranted.

**Fig. 2:**
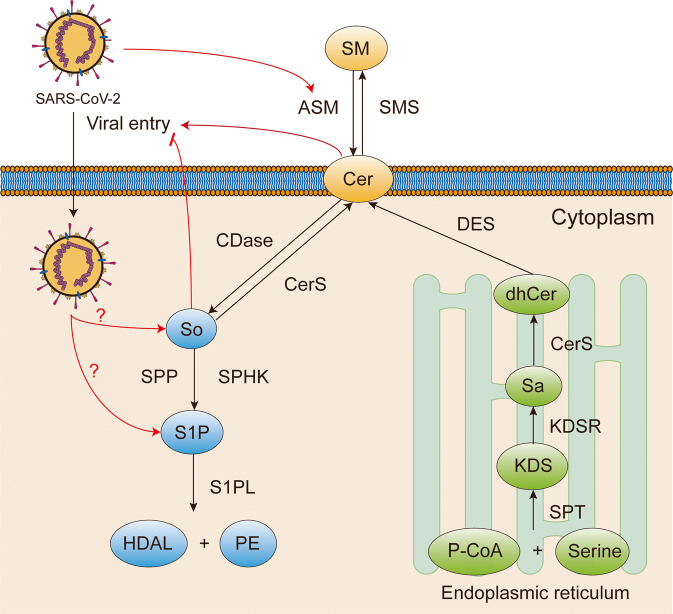
The sphingolipids metabolism pathway. Sphingomyelin on the plasma membrane can be converted to ceramide by sphingomyelinase. Ceramide can also be synthesized de novo from palmitoyl CoA and Serine or synthesized from sphingosine by ceramide synthase. SM Sphingomyelin, Cer ceramide, So Sphingosine, S1P Sphingosine 1-phosphate, HDAL Hexadecenal, PE Phosphorylethanolamine, dhCer dihydroceramide, Sa Sphinganine, KDS 3-ketodihydrosphingosine, P-CoA Palmitoyl-CoA, ASM Acid sphingomyelinase, SMS Sphingomyelin synthase, CDase Ceramidase, CerS Ceramide synthase, SPP S1P phosphatase, SPHK Sphingosine kinase, S1PL S1P lyase, DES dihydroceramide Δ4-saturase, KDSR 3-ketodihydroshpingosine reductase, SPT Serine palmitoyltransferase

Many viruses including ebola virus (EBOV), human immunodeficiency virus type I (HIV-1), hepatitis C virus (HCV), and simian virus 40 (SV40) are reported to employ *sphingolipids* for cell membrane attachment.^88–91^ The sphingolipid metabolism pathway is also manipulated for SARS-CoV-2 entry (Fig. 2). Sphingolipids and their metabolites together comprise a complex network of signaling in a series of physiological processes, including maintaining cellular structure, relaying signals, and modulating enzymatic activity.^92^ Elevated sphingolipid levels stimulated by SARS-CoV-2 were observed in cells and mice serum. Analysis of COVID-19 patient serum samples indicated a distinct alteration in sphingolipid profiles. The result shows a progressive increase in dihydrosphingosine, dihydroceramides, ceramides, sphingosine, and a decrease in sphingosine-1-phosphate (S1P).^93,94^ Acid sphingomyelinase (ASM) catalyzes the hydrolysis of sphingomyelin to ceramide and phosphorylcholine. Increased circulating activity of ASM and derangement of sphingolipids were observed in COVID-19 patients. The increase of ASM activity accurately distinguishes the patient cohorts undergoing intensive care from healthy controls.^94^ Among the various sphingolipids, the impact of ceramide and sphingosine on SARS-CoV-2 entry is prominent. Ceramide is converted from sphingomyelin by ASM or synthesized de novo from palmitoyl CoA and serine. Several ASM inhibitors including antidepressants (Amitriptyline, Imipramine, Fluoxetine, Sertraline, and Escitalopram) or ASM-knockout potently hindered SARS-CoV-2 entry in vivo. The suppressive effect is exerted partially via an impaired surface ceramide level since the replenishment of exogenous ceramide restored SARS-CoV-2 entry.^95^ Ceramide-enriched domain formed by released ceramide on the cell surface promoted SARS-CoV-2 entry.^96,97^ Furthermore, SARS-CoV-2 induced ACE2 clustering in ceramide-enriched domains on the membrane of nasal epithelial cells isolated from healthy donors. Ambroxol, an ASM inhibitor, potently suppressed ACE2 clustering on the cell membrane and reduced viral uptake by the epithelial cells.^98^ Fluoxetine, amiodarone and imipramine exhibited profound inhibitory activity on SARS-CoV-2 entry. This study indicated that further than removing membrane ceramide, ASM inhibitors can induce endolysosomal cholesterol accumulation and dysregulated acidification, hence blocking SARS-CoV-2 entry via the endosomal pathway.^99^ It is noteworthy that C16 ceramide presumably plays a central role in promoting SARS-CoV-2 entry since an exogenous supplement of C16 ceramide restored SARS-CoV-2 infection under ASM inhibitors treatment.^98^ The precise role of ceramide needs to be further defined.

***Sphingosine*** derives from ceramide by ceramidase catalyzation or from S1P by S1P phosphatase catalyzation. Sphingosine plays quite a contrary role to ceramide: while ceramide promotes SARS-CoV-2 entry, sphingosine impedes it. Sphingosine binds with membrane ACE2, thereby blocking the interaction between ACE2 and S protein, consequently inhibiting SARS-CoV-2 entry.^100^ The above findings provide reference for therapeutic interventions since drugs interfering with sphingolipids metabolism pathway like antidepressants are well-tolerated and extensively applied in clinic.^101^

***Glycolipids*** are essential for SARS-CoV-2 entry, especially sialylated glycolipids. Monosialylated gangliosides have a strong binding affinity with the receptor binding domain (RBD) of S protein. GENZ-123346 is an inhibitor of UDP-glucose ceramide glycosyltransferase (UGCG) that can deplete glycolipids from the cell membranes. GENZ-123346 treatment caused profoundly attenuated SARS-CoV-2 entry in vitro. Consistently, UGCG ablation by CRISPR/Cas9 also curbed SARS-CoV-2 infection.^102^ Chloroquine binds with sialic acids and gangliosides with a higher affinity than S protein. Treatment of chloroquine or its derivative, hydroxychloroquine, actively reduced SARS-CoV-2 S protein binding with gangliosides and exhibited potent antiviral activity.^103,104^ Moreover, chloroquine and hydroxychloroquine can be incorporated into endosomes, resulting in an increase of endosomal pH and prevention of viral entry via endocytosis.^105^ Results from in vitro experiments further confirmed the antiviral effect of chloroquine and hydroxychloroquine against SARS-CoV-2.^58^ Nevertheless, the clinical use of chloroquine or hydroxychloroquine is still controversial due to insufficient clinical data. Although some clinical studies showed the benefit of chloroquine and hydroxychloroquine, they are not completely reliable because they are non-peer-reviewed, unblinded, or non-randomized.^106^ Besides, the cardiotoxicity of chloroquine should also be taken into consideration since cardiovascular disorders are also major complications of COVID-19. The European Medicines Agency has refused to approve chloroquine for COVID-19. However, several clinical trials in China proved the efficacy of chloroquine and hydroxychloroquine.^107–109^ The use of chloroquine for the treatment of COVID-19 has been added to the guideline (version 6) in China.

Intriguingly, a recent report identified the bile acid receptor *farnesoid X receptor (FXR)* as a direct regulator of ACE2 expression. The presence of the FXR responsive element was uncovered in the ACE2 promoter region. Upon activation, FXR directly binds with the ACE2 promoter, confirmed by chromatin immunoprecipitation. Bile acid chenodeoxycholic acid (CDCA) is the major agonist of FXR. Treatment of CDCA markedly upregulated ACE2 expression and enhanced SARS-CoV-2 infection in vitro, in vivo, and in organoids in an FXR-dependent manner. A clinically approved FXR inhibitor, ursodeoxycholic acid (UDCA), significantly downregulated ACE2 expression and exhibited potent antiviral activity in vitro, in vivo, in organoids, and in human organs. In humans, UDCA treatment also decreased ACE2 levels in the nasal epithelium, a primary site for SARS-CoV-2 infection. The retrospective study also demonstrated that patients on UDCA were less likely to develop moderate and severe COVID-19. Altogether, this study demonstrates: (1) FXR directly regulates ACE2 expression; (2) ACE2 levels closely associate with SARS-CoV-2 entry; (3) UDCA could be used as a prophylaxis or a therapy for COVID-19.^110^

***Viral protein modifications*** are functionally essential in life cycles of coronaviruses. Targeting the post-translation modifications is promising.^111–113^ The lipid modification on S protein is indispensable in SARS-CoV-2 entry. Structural analysis reveals substantial conformational rearrangements of RBD during the infection process: a switch from a closed conformation to an open conformation. In a closed conformation, RBD is buried and less accessible for ACE2 binding; while in an open conformation, RBD exposes the receptor binding motif, enabling ACE2 binding.^114–117^ A hydrophobic pocket in S protein was detected, into which linoleic acid fits well. Although the linoleic acid binding pocket is distal from the receptor binding motif, linoleic acid binding results in stabilization of the closed conformation of S protein and compaction of homotrimer architecture, consequently reducing ACE2 binding and membrane fusion.^118^ Another computational simulation also showed that the presence of linoleic acid in S protein stabilizes the closed conformation and blocks its interaction with ACE2.^119^ Molecular dynamics simulation revealed that the linoleic acid binding site is coupled to functionally relevant regions of S protein. Removal of a ligand from the linoleic acid binding site disturbed the dynamics of distant functionally important regions of S protein.^120^

Several studies have reported that protein *palmitoylation* is crucial in host-virus interaction.^121–123^ Viral protein palmitoylation has a selective advantage among coronaviruses, and the palmitoylation sites are conservative.^122^ SARS-CoV-2 S protein contains a highly conserved free fatty acid binding pocket with unknown evolutionary selection advantage.^124^ Quite contrary to linoleic acid, S protein palmitoylation functionally promotes SARS-CoV-2 entry. Palmitoylation site C15 at the N terminus and other sites locating in the cytoplasmic tail of S protein have been identified by two independent studies.^125,126^ Zinc finger DHHC domain-containing palmitoyltransferase (ZDHHC) inhibitor reduces S protein palmitoylation, consequently decreasing S-mediated syncytia formation and SARS-CoV-2 pseudovirus entry.^125^ Mutation of palmitoylation sites in the cytoplasmic tail culminated in impeded SARS-CoV-2 pseudovirus entry. The inhibitory effect could be attributed to reduced S protein homotrimer stabilization by depalmitoylation.^126^ ZDHHC5 and GOLGA7 together form an acyl-transferase complex that mediates protein palmitoylation. The interaction between SARS-CoV-2 S protein and ZDHH5/GOLGA7 complex has been confirmed for S protein palmitoylation.^127^ Another study suggested that S-acyltransferase ZDHHC20 and 9 mediate the palmitoylation of SARS-CoV-2 S protein.^128^ Palmitoylation sites have also been observed in the cysteine-rich domain of SARS-CoV S protein, indicating the conservation of S protein palmitoylation.^129,130^

**Fig. 3:**
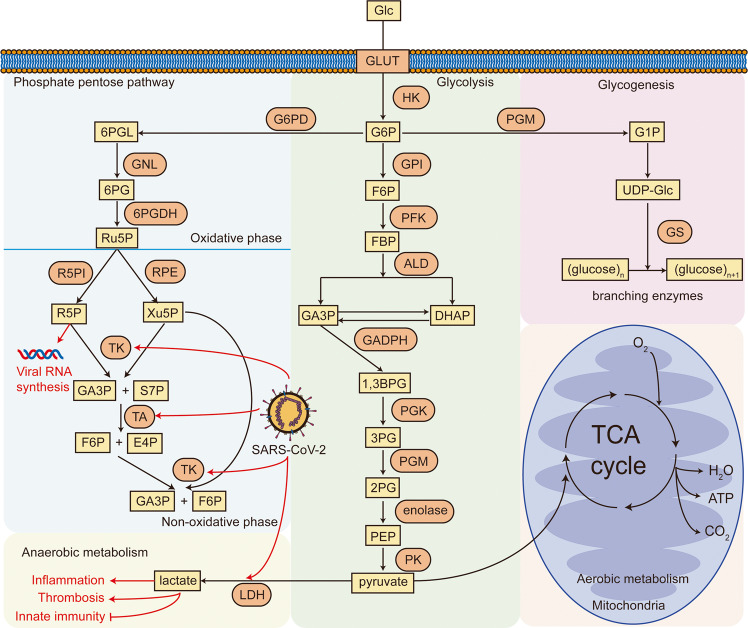
Major pathways of glucose metabolism. Glycolysis, glycogenesis, phosphate pentose pathway, aerobic metabolism, and anaerobic metabolism are included. GLUT glucose transporter, Glc glucose, G6P glucose-6-phosphate, F6P fructose-6-phosphate, FBP fructose-1,6-biphosphate, GA3P glyceraldehyde-3-phosphate, DHAP dihydroxyacetone phosphate, 1,3BPG 1,3-biphosphoglycerate, 3PG 3-phosphoglycerate, 2PG 2-phosphoglycerate, PEP phosphoenolpyruvate, G1P glucose-1-phosphate, UDP-Glc uracil-diphosphate glucose, 6PGL 6-Phosphogluconolactone, 6PG 6-phosphogluconate, Ru5P ribulose-5-phosphate, R5P ribose-5-phosphate, Xu5P xylulose-5-phosphate, S7P Sedoheptulose-7-Phosphate, E4P erythrose-4-phosphate, HK hexokinase, GPI glucose-6-phosphate isomerase, PFK phosphofructokinase, ALD aldolase, GADPH glyceraldehyde-3-phosphate dehydrogenase, PGK phosphoglycerate kinase, PGM Phosphoglycerate mutase, PK pyruvate kinase, GS glycogen synthase, G6PD glucose-6-phosphate dehydrogenase, GNL gluconolactonase, 6PGDH 6-phosphogluconate dehydrogenase, R5PI ribose-5-phosphate isomerase, RPE ribulose-5-phosphate 3-epimerase, TK transketolase, TA transaldolase, LDH lactate dehydrogenase

***High glucose*** levels promote SARS-CoV-2 entry by upregulating ACE2 expression. The major glucose metabolism pathways are shown in Fig. 3. In vitro assay demonstrated that high glucose levels significantly stimulated ACE2 overexpression in A549 cells.^131^ The correlation between elevated ACE2 expression and diabetes mellitus was also confirmed in mice models.^132^ A phenome-wide Mendelian randomization study revealed a significant correlation between elevated ACE2 expression and T2D.^133^ Furthermore, diabetes patients are often treated with ACE inhibitors and hypoglycemic drugs that can upregulate ACE2 expression.^134–137^ Notably, elevated ACE2 expression has been directly linked to reinforced viral entry. Increased ACE2 expression in lungs, kidneys, myocardium, and pancreas can promote SARS-CoV-2 binding.^138,139^ A recent study indicated that glucose treatment induced a dramatic upregulation of ACE2 expression in human kidney organoids, consequently promoting SARS-CoV-2 infection. More importantly, kidney cells from the biopsies of diabetic patients were more susceptible to SARS-CoV-2 infection compared to the kidney cells from healthy controls. From a mechanistic view, hyperglycemia increases the stability of ACE2 mRNA, partially explaining the ACE2 overexpression.^140^ Therefore, preexisting diabetes, hyperglycemia, and associated medications predispose patients to severe COVID-19 partially via promoting viral entry by upregulating ACE2 expression.

***Viral protein glycosylation*** plays critical roles in mediating protein folding, stabilization, interaction with host factors, and immune evasion.^111,141^ Beside lipid modifications, S protein of coronaviruses is also highly glycosylated.^142–144^ Twenty-two N-linked glycosylation sites and several O-linked glycosylation sites have been identified in SARS-CoV-2 S protein and confirmed by mass spectrometry. The glycan types of the O- and N-linked glycosylation have also been investigated.^145–149^ A high throughput study revealed that mutations at the glycosylation sites could significantly affect the infectivity of S protein pseudo-viruses. The mutations in glycosylation sites at RBD impaired viral infectivity by over 100-fold, suggestive of their functional significance. Furthermore, mutations in glycosylation residues affected the viral sensitivity to antibodies. N165Q mutation increased the sensitivity to neutralizing monoclonal antibodies, while N234Q mutation attenuated it.^150^ The importance of N-glycans at N165 and N234 in modulating conformational dynamics of S protein was also highlighted by a computational simulation.^151^ STT3A is a crucial glycosyltransferase that catalyzes N-glycosylation of S protein. N-linked glycosylation inhibitor 1 (NGI-1) inhibits STT3A to reduce S protein N-glycosylation. NGI-1 treatment dramatically decreased the binding ability of recombinant S protein to ACE2 and impaired the infectivity of SARS-CoV-2.^152^ Blockade of S protein N-glycosylation by genetic approaches or kifunensine (mannosidase-I alkaloid inhibitor) only had a mild impact on S protein-ACE2 binding, but markedly reduced pseudo-virus entry. It is well known that S1-S2 site cleavage is closely associated with viral entry.^153,154^ Interestingly, the inhibitory effect on reduced N-glycans is mediated through an enhanced S1-S2 site cleavage.^155^ O-glycans of S protein were also demonstrated to modulate S1-S2 site cleavage. UDP-Gal-NAc:polypeptide N-acetylgalactosaminyltransferases 1 (GALNT1) catalyzes O-glycosylation in residues proximal to the S1-S2 site. O-glycosylation near the cleavage site significantly impeded furin cleavage and syncytia formation. The O-glycosylation is dependent on P681. P681 mutation significantly decreased furin cleavage, O-glycosylation, and syncytia formation.^156^ In conclusion, both N- and O-glycosylation of S protein affect SARS-CoV-2 entry, partially via disturbing furin cleavage at the S1-S2 site. Targeting enzymes required for S protein glycosylation by inhibitors, such as 2-deoxy-d-glucose (2-DG) or NGI-1, are promising to be used for COVID-19 therapy.^157^

### Virus replication

SARS-CoV-2 reorganizes the ER network and alters the morphology of this organelle to generate viral replication compartments, which contain predominantly DMVs, but also incorporate ER connectors, double membrane spherules, and multi-membrane vesicles. Results from electron tomography showed that DMVs of SARS-CoV-2 are tethered to ER, and several DMVs are attached to the same ER branch, implicating the derivation of DMVs from ER.^22^ Several independent studies indicated that SARS-CoV-2 can usurp certain stages of the autophagy pathway for DMV formation. Class III phosphatidylinositol 3-kinase (PI3K) mediates autophagosome formation in the autophagy pathway. SARS-CoV-2 uses PI3K to produce phosphatidylinositol 3-phosphate (PI3P) for DMV formation, and PI3K inhibition or genetic depletion profoundly impaired DMV formation and viral replication.^158^ Several SARS-CoV-2 viral proteins are proposed to manipulate the autophagy pathway. ORF7a reduces lysosome acidity to prevent autophagosome degradation. ORF3a blocks the fusion between lysosomes and autophagosomes, consequently causing the retention of autophagosomes.^159,160^ The degradation of autophagy-initiating protein Beclin-1 (BECN-1) induces the fusion of autophagosomes with lysosomes, and this process is regulated by Akt/SKP2 pathway.^161^ In lung samples of COVID-19 patients, phagophore-incorporated autophagy markers LC3B-II and p62 were detected, indicating autophagosome accumulation, which can be attributed to the impact of SARS-CoV-2 on BECN1 levels through AKT1/SKP2 signaling.^162^ Research on other coronaviruses also underlines the importance of the autophagy pathway in viral DMV formation.^163,164^ In DMVs, viral RNA and NSPs assemble into RTCs and trigger RNA synthesis.^165^

Diverse metabolism processes, including fatty acid oxidation, cholesterol catabolism, pentose phosphate pathway, one carbon metabolism, and fatty acid synthesis, are manipulated to fulfill high substrate and energy demand for the replication of (+) ssRNA viruses.^30,166–168^

***Phosphatidic acid*** (PA) is generated by acylglycerolphosphate acyltransferase 1 and 2 (AGPAT1/2) in the ER. It was postulated that PA subverts ER membrane to form replication organelles of SARS-CoV.^169^ During SARS-CoV-2 infection, AGPAT1/2 were recruited to RTCs and critically contribute to SARS-CoV-2 replication and DMV formation. Pharmacological inhibition of PA synthesis impaired autophagosome-like DMV formation.^170^ Phosphatidic acid phosphatase 1 (PAP1) converts PA to diacylglycerol, a substrate for phosphatidylethanolamine (PE) and phosphatidylcholine (PC) synthesis.^171^ Intriguingly, inhibition of PAP1 activity suppressed SARS-CoV-2 replication. PE and PC are essential for (+) ssRNA viral replication via driving replication organelle formation.^172–176^ Thus, inhibition of PAP1 or AGPATs likely curbs SARS-CoV-2 replication via reducing cellular downstream PE and PC levels.

***Fatty acids*** are also essential for SARS-CoV-2 replication. Pharmacological inhibition of fatty acid synthase impaired SARS-CoV-2 replication, which was restored by exogenously supplied fatty acids.^177,178^ The exploitation of fatty acids was also seen in Dengue virus infection. NSP3 of Dengue virus recruits fatty acid synthase (FASN) to the replication site and profoundly activates FASN. The author suggests that DENV could co-opt host FASN to establish its replication complexes.^179^ However, available studies on the exact role of fatty acids in SARS-CoV-2 replication are lacking.

***Lipid droplets*** play a crucial role in viral replication of different viruses including coronaviruses. Disrupting lipid droplets could be a promising therapeutic strategy.^35,180,181^ Lipid droplets were considered mere lipid storages for a long time, but current findings expand their functions also as central hubs for lipid homeostasis regulation and as effectors in viral infection.^168,182,183^ Lipid droplets are mainly composed of triacylglycerols, cholesteryl esters, and various enzymes for lipid metabolism, surrounded by a phospholipid monolayer.^184,185^ Lipid droplet generation is regulated by several enzymes. FASN yields fatty acids, which are further converted to diacylglycerols, and then to triacylglycerols by diacylglycerol o-acyltransferase 1 and 2 (DGAT1/2). Key enzymes responsible for cholesterol synthesis in lipid droplets are acyl-CoA cholesterol acyltransferase 1 and 2 (ACAT1/2).^186,187^ Since lipid droplets are involved in the replication of many RNA viruses, it is postulated that they probably affect SARS-CoV-2.^188^ Lipid droplets are found to be located in close proximity to intracellular SARS-CoV-2 particles. In addition, A 922500, a DGAT-1 inhibitor, decreased viral load and ameliorated cytopathy.^189^ DGAT1/2 and lipid droplet stabilizer adipocyte differentiation-related protein (ADRP) can assist SARS-CoV-2 replication. SARS-CoV-2 N protein is demonstrated to upregulate DGAT1/2 expression to generate lipid droplets. N protein interacts with ADRP on the lipid droplet surface to favor viral replication.^190^ Peroxisome proliferator-activated receptor-α (PPAR-α) induces lipid droplet degradation by β-oxidation. Palmitoylethanolamide (PEA), a PPAR-α agonist, significantly suppressed SARS-CoV-2 replication by decomposing lipid droplets.^191^ NSP6 was proposed to mediate the contact of SARS-CoV-2 replication organelle with lipid droplets by recruiting the lipid droplet-tethering complex DFCP-1.^192^

***TMEM41B***, an ER-localized protein, has been identified as a mediator for lipid mobilization from lipid droplets.^193,194^ TMEM41B is a paramount host factor with diverse functions in coronavirus replication.^195,196^ Two independent genome-wide screens identified TMEM41B as an important host factor for SARS-CoV-2 replication. Inhibition or genetic ablation of TMEM41B attenuated SARS-CoV-2 replication in an early post-entry stage. Although TMEM41B is involved in the autophagy pathway,^197,198^ the impact of TMEM41B on lipid localization and trafficking from lipid droplets mainly accounts for assisting viral replication since knockout of TMEM41B did not compromise SARS-CoV-2 replication via suppressing the induction of autophagosomes.^199,200^ Collectively, the above studies suggest that TMEM41B mediates the lipid exchange between SARS-CoV-2 replication organelle and lipid droplets. In a genome-wide CRISPR/Cas-9 knockout screen, TME41B was also identified as a conserved host factor required for replication among coronaviruses.^201^

It is well-known that SARS-CoV-2 infection also causes a shift of ***glucose metabolism*** to aerobic glycolysis, namely the Warburg effect.^202–204^ Most viruses including coronaviruses induce Warburg effect in favor of their replication.^205^ Glycolysis provides energy and building blocks for nucleotide synthesis, which is conducive to viral RNA replication. Other viruses are reported to hijack glycolysis for RNA and protein synthesis.^205,206^ Metabolomic analysis showed lower intracellular glucose levels and higher lactate levels at early time points after SARS-CoV-2 infection, suggestive of enhanced glycolysis to meet the massive demand for viral replication.^207^ Proteomics study revealed an increased protein cluster of carbon metabolism during SARS-CoV-2 infection, and blockade of glycolysis by 2-DG prevents SARS-CoV-2 replication.^208^ It was also reported that SARS-CoV-2 triggered ROS production to stabilize HIF-1α, culminating in the Warburg effect.^209^ Moreover, the blockade of HIF-1α by GW6471 effectively inhibited SARS-CoV-2 infection in airway organoids.^210^

The ***Warburg effect*** leads to an increase in the activity of hexokinase (HK), which is the first rate-limiting enzyme of glycolysis. HK catalyzes the generation of glucose-6-phosphate, the primary material for the pentose phosphate pathway (PPP). The PPP generates NADPH and ribose-5-phosphate to provide material and energy for a variety of cellular events.^211,212^ Glucose-6-phosphate dehydrogenase (G6PD) is an important enzyme in PPP that converts glucose-6-phosphate to 6-phosphogluconolactone. G6PD was found to be upregulated in lungs obtained from deceased COVID-19 patients.^213^ The PPP was also proposed to be involved in SARS-CoV-2 replication to meet the high demand for ribose-5-phosphate for viral RNA synthesis. SARS-CoV-2 infection upregulated the levels of two constituents in the non-oxidative PPP branch: transketolase and transaldolase 1. Benfooxythiamine, a transketolase inhibitor, significantly curbed SARS-CoV-2 replication. 2-DG interferes with glycolysis to reduce the material for the PPP. The combination of 2-DG and benfooxythiamine synergistically restrained SARS-CoV-2 replication, indicating that PPP inhibition and glycolysis inhibition can independently restrain SARS-CoV-2 replication.^214^

**Fig. 4:**
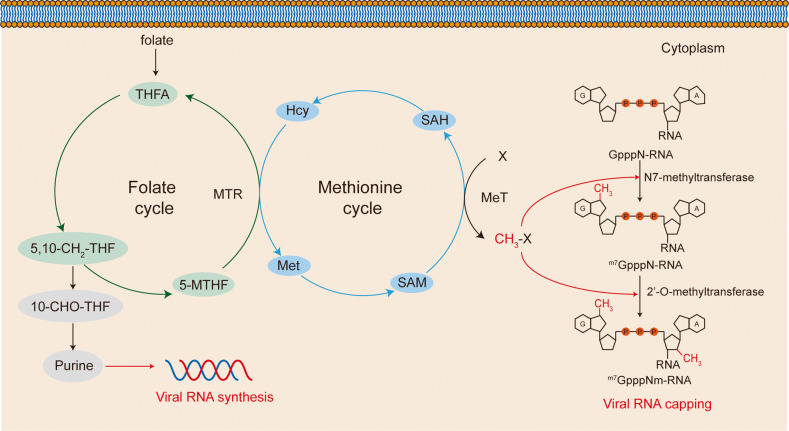
One carbon metabolism. One carbon metabolism mainly contains the folate cycle and the methionine cycle. SAM provides methyl for methylation, then it is recycled by the methionine cycle. Folate drives the folate cycle to mediate transfer of one carbon unit and purine de novo synthesis. THFA tetrahydrofolate, 5-MTHF 5-methyltetrahydrofolate, 10-CHO-THF 10-formyltetrahydrofolate, 5,10-CH2-THF 5,10-methylene tetrahydrofolate, Hcy homocysteine, Met methionine, SAH S-adenosylhomocystein, SAM S-adenosylmethionine, MeT methyltransferase, MTR methionine synthase

***Folate*** is required for one-carbon unit transfer, which is essential for purine de novo synthesis. The one-carbon metabolism is shown in Fig. 4. Folate species convey one carbon unit to mediate de novo purine synthesis.^215,216^ Metabolomic analysis detected the accumulation of intermediates of de novo purine synthesis in SARS-CoV-2-infected cells. Intracellular glucose and folate were found to be depleted during the infection of SARS-CoV-2, indicative of the consumption of them. Methotrexate is a folate analog that disrupts one carbon pathway. Treatment of methotrexate markedly reduced SARS-CoV-2 RNA levels, viral protein expression, and virion production, highlighting the importance of one carbon pathway and de novo purine synthesis in SARS-CoV-2 replication.^207^ Notably, methotrexate is FDA-approved for inflammatory disorders with a good safety profile. Thus, methotrexate is prospective to be applied to SARS-CoV-2 therapy.

***One carbon metabolism*** also affects SARS-CoV-2 replication by boosting S-adenosylmethionine (SAM)-dependent methylation. RNA capping plays a key role in viral replication by evading the immune system, maintaining RNA stability, and initiating translation.^217^ RNA capping machinery is employed by different species of coronaviruses.^218–220^ SARS-CoV-2 also employs RNA capping. In SARS-CoV-2, the RNA cap is composed of a 7-methylguanosine linked to the 5’ nucleotide of the viral RNA through a triphosphate bridge. The cap is methylated at the N7 site of the guanosine, using SAM as a methyl donor, forming ^m7^GpppN-RNA, mediated by NSP14.^221^ Then, SAM-dependent 2’-O-methyltransferase adds a methyl group to the ribose 2’-O site of the nucleotide to consequently form the cap (^m7^GpppNm-RNA), mediated by NSP16.^222^ Folate metabolism fuels SAM production by maintaining the methionine cycle. The methionine cycle is essential for the generation of SAM through the adenylation of methionine.^223^ SAM cycle inhibitors or genetic ablation of main enzymes in the SAM cycle were shown to dramatically restrict SARS-CoV-2 replication. More importantly, 3-deazaneplanocin A, an FDA-approved drug that can disrupt SAM production, exhibited excellent antiviral potency.^224^

### Virion assembly

SARS-CoV-2 assembly occurs in the ERGIC.^27,29^ Structural proteins are essential for this process. After replication, newly synthesized RNA exits DMVs through molecular phores.^25^ A protein inside DMVs, probably an RNA-dependent RNA polymerase (RdRp), is hypothesized to guide the viral RNA through these pores. Viral RNA is subsequently condensed by N protein.^225^ The N-RNA complex is organized to form G-shape ribonucleoproteins (RNPs). During assembly, N protein induces N-RNA complex phase separation via its central disordered domain.^225^ SARS-CoV M protein interacts with N protein via their highly hydrophilic and charged carboxy groups, overlaying the N-RNA complex into the particle.^226,227^ Despite functional studies on SARS-CoV-2 M protein are still blank, considering the high homology between SARS-CoV M protein and SARS-CoV-2 M protein, SARS-CoV-2 M protein possibly incorporates N-RNA complex with the same strategy.^228^ The interaction between E and M in coronaviruses is well-established. Co-expression of the M and E proteins is sufficient for virion maturation and release.^229–231^ E and M interacts with each other via the C-terminal domain of both proteins at the cytoplasmic side of ERGIC.^105,232^ E protein is able to oligomerize to form a pentamer and serves as a cation channel to increase pH in ERGIC.^233^ S is considered less involved in the assembly process since viral assembly without S is not affected.^229^ S protein clusters through M-S interaction when the N-RNA complex is incorporated into a newly formed particle.^29^ M is indispensable for S incorporation into virions.^227^ Both M and E induce maturation and S location in ERGIC.^234^

The process of viral release is one of the least understood stages in the life cycle of SARS-CoV-2. Previously, it was a mainstream theory that coronaviruses primarily egress through the biosynthetic secretory pathway. However, a recent finding from transmission electron microscopy proposes that coronavirus including SARS-CoV-2 more likely egresses in small secretory vesicles.^235^ Another study proposes a model in which coronaviruses egress by lysosomes instead of the biosynthetic secretory pathway. The lysosomes were deacidified with proteolytic enzymes inactivated in cells infected by SARS-CoV-2.^28^ The mechanism of SARS-CoV-2 release remains a question of debate. Hence, there are few available studies connecting viral exit and host lipids.

Cellular lipids play crucial roles in multiple processes of SARS-CoV-2 assembly. M binds with lipid headgroups through their C-terminus domain. When M homodimer is in an open conformation, cholesterol and inositol lipids accumulate in the vicinity of the homodimer, while phosphatidylserines accumulate near a monomer of the homodimer. The author suggests that the lipid-protein interplay explains membrane curvature formation induced by M protein.^236^

E protein is a functional membrane morphogen. When synthesized alone, E reconfigures intracellular membranous organelles into elongated swirls.^237^ Palmitoylation of coronavirus E protein is functionally important in viral assembly.^238–240^ Palmitoylation of E protein occurs in a conserved cysteine-rich region near the transmembrane domain, which is primarily catalyzed by the DHHC-rich domain palmitoyl acyltransferase that resides in ER and Golgi. These integral membrane proteins transfer palmitate residing in the DHHC motif to the cysteine residues in the acceptor proteins.^241,242^ Mouse hepatitis coronavirus (MHC) E protein has three conserved cysteine residues in their cysteine-rich regions. Replacement of cysteine residues in MHC E protein by alanines or glycines culminated in failed virus-like particle secretion and disturbed virion assembly when expressed along with N, M, and S protein.^240^ Triple cysteine/alanine mutations in the cysteine-rich region led to significantly reduced MHC virus yields in the infected cells. Furthermore, MHC E protein lacking all three cysteines exhibited an increased rate of degradation, indicating a compromised stability.^243^ In SARS-CoV, E also undergoes intracellular post-translational modifications. Palmitoylation of SARS-CoV E protein is functionally essential for maintaining stability.^244^ Since the sequence of SARS-CoV-2 E protein is 95 % identical to the counterpart of SARS-CoV, SARS-CoV-2 E protein palmitoylation probably has a similar function. Molecular dynamic simulations on SARS-CoV-2 E protein indicate that palmitoylated E protein is more stable. In the absence of palmitoylation, the pentameric structure of E proteins lost dynamic equilibrium, and the pore radius of E protein drastically decreased and even collapsed.^245,246^ Since both S and E proteins require palmitoylation to maintain their functional architecture, inhibitors targeting palmitoyl acyltransferase are of interest.

### Pathogenesis

Common symptoms of COVID-19 are mild, including fever, diarrhea, and cough, accounting for ~70% of patients.^247^ However, some patients progressed to more severe syndromes. Manifestations of severe COVID-19 patients are predominantly ARDS, thrombotic complications that mimic disseminated intravascular coagulopathy, and multi-organ failure. The pathology of COVID-19 is complicated and not fully elucidated, involving disruption of immune balance, systemic inflammation, complement hyperactivation, etc.^3,248^

An abnormal elevation of pro-inflammatory cytokines (IL-2R, IL-6, IL-8, TNF-α, et al.) and chemokines (CCL2, CCL8, CXCL9, et al.), together with an altered lymphocyte subset profile was observed in COVID-19 patients, reflecting the onset of autoimmune-related disorders.^249,250^ Intracellular innate immunity is also disrupted by SARS-CoV-2 for immune evasion.^251^ Interferon I-III (IFN I-III), nuclear factor-κB (NF-κB), toll-like receptor 4 (TLR4), retinoic-acid inducible gene I (RIG-1)-like receptor (RLR)/mitochondria anti-virus signaling protein (MAVS) pathways are all reported to be disturbed by SARS-CoV-2 with concomitant dysfunctional antiviral immunity and abnormal cytokine secretion.^252–259^ In extreme cases, the immune system over-reacts to the virus and triggers a fatal cytokine storm which is characterized by uncontrolled excessive pro-inflammatory cytokine release and hyperactivated immune cells. In a cytokine storm, immune cells erroneously attack normal host cells, causing collateral multi-tissue damages and multi-organ failure, bringing poor prognosis and even mortality.^260–262^ A cytokine storm was suggested as a major cause of lung damage in COVID-19.^263–265^ Coagulopathy in COVID-19 patients was evidenced by significantly elevated levels of D-dimer (a biomarker of coagulopathy) and thrombocytopenia in hospitalized patients.^7^ A large proportion of severe COVID-19 patients obtained a hypercoagulable state that predisposed patients to thrombosis. The Post-mortem of lung tissues revealed thrombogenesis in small arteries and capillaries of the pulmonary vasculature.^266–268^ The etiology of thrombosis in COVID-19 is under investigation. Coagulopathy, complement activation, pro-inflammatory cytokine release, platelet hyperactivation, and endothelial dysfunction are emerging as potential major contributors to thrombosis.^269^ Several compounds that ameliorate inflammation response have been demonstrated to alleviate symptoms of COVID-19. Dexamethasone is a corticosteroid widely used to treat a range of infections by resolving inflammation and suppressing abnormally elicited immune responses.^270^ In patients who need ventilation, dexamethasone decreased the mortality rate by approximately one-third.^271^ Baricitinib is a reversible JAK1/2 inhibitor that regulates inflammation and immune responses. In hospitalized COVID-19 patients, treatment with Barcitinib effectively decreased the mortality rate.^272^ Given that host lipids have immunomodulatory properties, they are supposed to be involved in SARS-CoV-2-elicited disorders. This section focuses on the role of host metabolism in the pathogenesis of COVID-19.

**Fig. 5:**
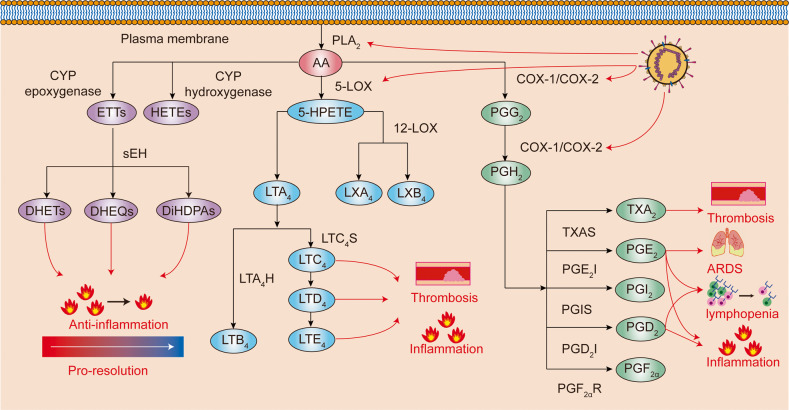
Arachidonic acid metabolism. The arachidonic metabolism pathway includes three branches: 1. CYP enzymes convert arachidonic acids to ETTs or HETEs. 2. 5-lipoxygenase catalyzes arachidonic acids to lipoxins and leukotrienes 3. The COX-1/COX-2 pathway that produces prostaglandins. AA arachidonic acids, PGG2 prostaglandin G2, PGH2 prostaglandin H2, TXA2 thromboxane A2, PGE2 prostaglandin E2, PGI2 prostaglandin I2, PGD2 prostaglandin D2, PGF2α prostaglandin F2α, 5-HPETE 5-hydroperoxyeicosatetraenoic acid, LXB4 lipoxin B4, LXA4 lipoxin A4, LTA4 leukotriene A4, LTB4 leukotriene B4, LTC4 leukotriene C4, LTD4 leukotriene D4, LTE4 leukotriene E4, ETTs epoxyeicosatrienoic acids, HETEs hydroxyeicosatetraenoic acids, DiHPAs dihydroxydocosapentaenoic acids, DHEQs dihydroxyeicosatetraenoic acids, DHETs dihydroxyeicosatrienoic acids, PLA2 Phospholipase A2, COX-2 cycloxygenase-2, COX-1 cycloxygenase-1, 5-LOX 5-Lipoxygenase, 12-LOX 12-Lipoxygenase, LTC4S LTC4 synthase, LTA4H LTA4 hydrolase, TXAS Thromboxane A synthase, PEG2I PEG2 isomerase, PGIS prostaglandin I synthase, PGD2 I PGD2 isomerase, PGF2αR PGF2α reductase, CYP cytochrome P450, sEH soluble epoxide hydrolase

***Eicosanoids*** are inflammation mediators originated from the processing of arachidonic acid (AA). AA is generated by the cleavage of membrane phospholipids by phospholipase A_2_ (PLA_2_). AA can be processed by cyclooxygenases (COX) or lipoxygenases (LPX) to produce prostaglandins (PGs), thromboxanes (TXs), or leukotrienes (LTs), which all play regulatory roles in immune homeostasis (the AA metabolism pathway is shown in Fig. 5). Analysis of bronchoalveolar lavage fluid of COVID-19 patients showed elevated PGE_2_, TXB_2_, 12-HHTrE, and leukotriene B_4_ (LTB_4_) levels compared to healthy controls, and the eicosanoid levels were positively correlated with the levels of cytokines (IL-1α, IL-6, TNF-α, IL-12p70, IL-22, and IFN-α2) and chemokines (CCL2, CCL11, CXCL9, and CXCL10).^273^

In hospitalized patients, plasma *PLA*_***2***_ levels positively correlated with disease severity and acute multisystem inflammatory syndrome.^274^ Lipidomic analysis on plasma samples of COVID-19 patients also identified PLA_2_ as an indicator of severity. Deceased patients had higher levels of PLA_2_ compared with survivors.^275^ The correlation could be attributed to downstream products of AA metabolism.

COX-1 and COX-2 catalyze AA to prostaglandin H_2_, which is further converted to diverse bioactive *prostaglandins (PGs)*. PGE_2_, the most abundant PG, is an important mediator of a series of physiological processes, especially initiation and regulation of inflammation.^276^ SARS-CoV-2 can stimulate COX-2 overexpression in diverse human cell lines.^277^ Risk factors for severe COVID-19 courses include obesity, older ages, and a sedentary lifestyle, and all these risk factors positively correlated with patient serum PGE_2_ levels. Clinical research revealed that in COVID-19 patients, high serum PGE_2_ levels are associated with poor prognosis. The increased COX-2 levels were observed in living human precision-cut lung slices, implying the causal relationship between ARDS and PGE_2_. Lymphopenia characterized by a drastic reduction of lymphocytes is generally accompanied by a poor prognosis of COVID-19 patients.^278^ PGE_2_ can suppress PAX5, a regulator of B cell proliferation and survival, thereby partially contributing to the lymphopenia.^279^ A comparatively low level of PGE_2_ enhances immunity while a high level of it compromises the immune system by killing lymphocytes.^280^ Another prostaglandin PGD_2_, synthesized by COX, also contributes to lymphopenia. PGD_2_ production can be upregulated by SARS-CoV infection, which subsequently disturbs lymphocyte priming and maturation via DP1 and DP2 signaling.^281^ The physiological function of PGE_2_ and PGD_2_ are double-edged and dose dependent. Both PGE_2_ and PGD_2_ can induce a lipid mediator class switching of eicosanoid production by neutrophils from the 5-lipoxygenase pathway to lipoxins, resolvins, and protectins.^282^ Hence, COX inhibitors should be used with caution.

Another COX downstream product *thromboxane A*_***2***_ *(TXA*_*2*_*)* also accounts for COVID-19 pathogenesis. TXA_2_ is synthesized from PGH_2_ predominantly by platelet COX. TXA_2_ induces platelet aggregation and thrombi generation. In COVID-19 patients, P-selectin, a marker of platelet activation expressed on the surface of platelet, is overexpressed. Plasma thrombopoietin levels are also upregulated, reflecting hyperactive platelet. Platelet extracted from COVID-19 patients aggregated faster and exhibited increased spreading on both fibrinogen and collagen. Otherwise, both circulating platelet-neutrophil and platelet-monocyte aggregates increased in COVID-19 patients, reflecting platelet hyperactivation and thrombogenesis. The platelet hyperactivation can be partially explained by MAPK activation and increased TXA_2_ production.^283,284^

Leukotrienes are produced from AA by 5-lipoxygenase. *Leukotriene A*_***4***_ *(LTA*_*4*_*)* is generated directly from AA, then converted to LTB_4_ in neutrophils and monocytes. Alternatively, LTA_4_ can also be converted to leukotriene C_4_ (LTC_4_) by LTC_4_ synthase. Outside the cell membrane, LTC_4_ is catalyzed to leukotriene D_4_ (LTD_4_) and leukotriene E_4_ (LTE_4_). LTC_4_, LTD_4_ and LTE_4_ are termed cysteinyl-leukotrienes.^285^ Leukotrienes act as chemotactic factors for the recruitment of lymphocytes, monocytes, and macrophages to peripheral tissues and are intimately related to the pro-inflammatory function of monocytes and macrophages.^286–288^ Considering the high neutrophil infiltration and high neutrophil/lymphocyte ratios in tissues of COVID-19 patients, leukotrienes are proposed to be involved.^289^ Moreover, single-cell analysis of bronchoalveolar immune cells and PBMCs from COVID-19 patients shows elevated 5-lipoxygenase expression.^290,291^ Leukotrienes also trigger cytokine (TNF-α, IL-1, IL-6, CCL2, etc.) release in the microenvironment to amplify inflammatory responses.^292,293^ Cysteinyl-leukotrienes activate platelet by binding with cysteinyl-leukotrienes receptors on platelet, culminating in thromboxane release and consequent coagulopathy as well as lung immunopathology in COVID-19.^248,294,295^ Besides, leukotrienes provoke atherosclerosis, plasma leakage, and ARDS, which are canonical manifestations of severe COVID-19.^288,296^ Lipidomic analysis of bronchoalveolar lavage fluid extracted from COVID-19 patients who require mechanical ventilation shows significantly elevated leukotrienes levels, predominantly LTB_4_, LTE_4_, and eoxin E_4_, supporting the deleterious role of leukotrienes.^297^ However, there are scarce studies on the precise role of leukotrienes in COVID-19 due to the difficulty in measuring leukotriene levels since they undergo rapid physiological metabolism. Of note, inhibition of COX could lead to an imbalance of AA metabolism and provoke leukotriene production, which partially explains why NSAIDs are of little benefit to severe COVID-19 patients.

Despite the benefit of inhibiting AA metabolism, inhibition of PLA_2_ may also downregulate pro-resolving factors which ameliorate inflammation. AA is a substrate for another enzymatic pathway, the cytochrome P450 (CYP) system. CYP system consists of two metabolic branches: ω-hydroxylases which convert AA to hydroxyeicosatetraenoic, and epoxygenases which convert AA to regioisomeric epoxyeicosatrienoic acids.^298^ Epoxyeicosatrienoic acids accelerate the termination of inflammation responses by mediating an array of anti-inflammatory and pro-resolving processes.^298,299^ Epoxyeicosatrienoic acids mitigate the release of pro-inflammation cytokines and chemokines, including IL-6, IL-1β, and MCP-1.^300^ In vivo, epoxyeicosatrienoic acids are constantly hydrated to dihydroxyeicosatrienoic acids by soluble epoxide hydrolase (sEH). Hence, inhibiting sEH can effectively upregulate epoxyeicosatrienoic acids levels. TPPU, an inhibitor of sEH, is able to block neutrophil infiltration to the lung, decrease pro-inflammatory cytokine levels in serum and bronchoalveolar lavage fluid, and ameliorate alveolar capillary leakage in an LPS-induced acute lung injury mouse model.^301^ Thus, although PLA_2_ inhibitors theoretically ameliorate COVID-19 by reducing downstream pro-inflammatory mediators, they possibly also hinder pro-resolution and anti-inflammation processes, consequently exacerbating the condition.

Besides AA metabolism, *ASM/ceramide* system also mediates inflammation and thrombogenesis during SARS-CoV-2 infection. In patients with community-acquired pneumonia, serum phospholipid levels greatly plunged, while ceramide levels increased and ASM activity was consistently enhanced.^302^ Sphingolipids are intimately associated with inflammation. In a mouse model, genetic ablation or pharmacological inhibiting ASM activity profoundly reduced pro-inflammatory cytokine production.^303^ Another study evaluated ASM serum activity in a mixed intensive care unit (ICU) population. Higher serum ASM levels in non-survivors and lower ASM levels in survivors were observed, and correlated with the mortality rates in ICU patients with systemic inflammation.^275^ In endothelial cells stimulated with serum from sepsis patients, plasma ASM activity and levels were profoundly enhanced, leading to endothelial stress response and cytotoxicity. However, pharmacological or genetic inhibition of ASM improved the sepsis-induced endothelial stress.^304^ More importantly, ASM also works as a pathogenic mediator of ARDS.^305^ Severe COVID-19 is usually accompanied by a hypercoagulable state, and tissue factors are the primary cellular initiator of coagulation.^306,307^ In common conditions, tissue factors remain cryptic, and sphingomyelin can maintain tissue factors in the encrypted state. However, hydrolysis of sphingomyelin by ASM activates tissue factors, culminating in coagulation. SARS-CoV-2 spike protein pseudovirus markedly enhanced the procoagulant ability of tissue factors, while inhibition or silencing ASM attenuated SARS-CoV-2 pseudovirus-induced tissue factor activation.^308^ Given that ASM activation plays dual roles in both viral entry and pathogenesis, ASM inhibitions are supposed to alleviate COVID-19 severity.^309^

Besides viral entry, the dysregulated *cholesterol metabolism* also causes deterioration of COVID-19 through modulating inflammatory response. It is a prevalent phenomenon that patients with lower pre-infection HDL levels underwent severe COVID-19 with higher mortality rates. Cholesterol, HDL, and LDL levels are lower in patients who underwent severe COVID-19 compared to patients with non-severe COVID-19.^310–313^ Cholesterol is closely associated with immunometabolism. Cholesterol accumulation in macrophages and other immune cells promotes inflammation by amplification of Toll-like receptor (TLR) signaling, inflammasome activation, and production of monocytes and neutrophils.^314,315^ Cholesterol trafficking to ER can activate NLRP3 inflammasome, a key mediator of infection-induced inflammation, consequently promoting inflammation and pro-inflammatory cytokine secretion. Treatment with statins abrogated NLRP3 inflammasome assembly and IL-1β secretion.^316,317^ Cellular cholesterol levels are mainly monitored in ER membrane by liver X receptor (LXR), Sterol regulatory element binding protein-2 (SREBP2), and erythroid 2 related factor1 (NRF1).^318^ When the cholesterol content in ER decreases, SREBP-2 migrates from ER to Golgi, then it is activated and translocated into the nucleus to trigger downstream transcription.^319^ SREBP-2 serves as a hub to connect cholesterol levels to immune response. Mechanistically, upon detecting the decreased intracellular cholesterol level, SREBP-2 associates with NLRP3 to form a tertiary structure that translocates to Golgi to facilitate inflammasome assembly.^320^ Furthermore, SREBP-2 interplays with NF-κB to mediate inflammation.^321,322^ Evidence supports that SARS-CoV-2 manipulates SREBP-2 to stimulate cytokine storm. SREBP-2 was highly elevated in the PBMCs of COVID-19 patients, and the mRNA levels of SREBP-2 positively correlated with disease severity. In addition, SREBP-2 C-term, the cleaved form of SREBP-2, significantly increased in the serum of severe and deceased COVID-19 patients. Treating with Fatostatin A, an SREBP-2 processing inhibitor, effectively suppressed pro-inflammatory cytokine production in the PBMCs of COVID-19 patients. Fatostatin A can also protect human umbilical vein endothelial cells (HUVEC) from LPS stimuli, indicative of the vascular protective function.^323^ It is interesting to interrogate whether circulating SREBP-2 C-term directly contributes to COVID-19 progression or not. Of note, SARS-CoV-2 infection mitigates intracellular cholesterol levels. SARS-CoV-2 likely reduces the intracellular cholesterol to activate SREBP-2.

***HDL*** is mainly responsible for the cellular efflux of cholesterol. By promoting cholesterol efflux, HDL reduces cholesterol accumulation in immune cells.^324^ HDL is proposed to have anti-inflammatory, antioxidant, antiviral, anti-coagulant, and vascular protective properties, working as pathogen scavengers that potentially involves in the removal of infectious material. In addition, HDL prevents infection of various DNA and RNA viruses by neutralization.^325–327^ HDL levels negatively correlated with COVID-19 severity and decreased with the deterioration of patients’ conditions, indicating that high HDL levels prevent severe COVID-19. In spite of the close association between HDL and COVID-19 severity, detailed studies on the interplay between COVID-19 and HDL are scarce. The relationship between cholesterol metabolism and immunometabolism is intertwined, and the cellular cholesterol level alteration during COVID-19 is dynamic and not fully interpreted. Better comprehension will optimize the use of statins for the treatment of COVID-19.

The association between COVID-19 and dysregulated *glucose metabolism* is bidirectional. Pre-existing T2D or hyperglycemia are identified as risk factors for severe COVID-19 with high mortality rates.^328,329^ On one hand, glucose metabolism facilitates SARS-CoV-2 entry and replication. On the other hand, SARS-CoV-2 infection worsens the situation of T2D or even mediates the onset of new type 2 diabetes.^204^ The interplay between dysregulated glucose profiles and poor outcomes in COVID-19 patients is complicated and not fully known. However, SARS-CoV-2 can impair islet function by directly infecting pancreatic cells. Human pancreatic cells express SARS-CoV-2 entry receptors including ACE2, TMPSSR2, and neuropilin-1 (NRP-1). SARS-CoV-2 infection can restrict insulin secretion and induce β cell apoptosis in vitro.^330^ SARS-CoV-2 also infects the induced pluripotent stem cell (iPSC)-derived pancreatic cultures containing endocrine and exocrine cells and provokes inflammatory responses. Of note, the autopsy of patients who died of COVID-19 confirms the SARS-CoV-2 infectivity of the pancreas.^331^ Approximately 17% of severe COVID-19 patients display an increased level of amylase and lipase (two biomarkers of pancreas injury), indicative of the caused pancreatic injury.^332^ It is also proposed that the usage of steroids during the treatment might contribute to the onset of hyperglycemia and diabetes.^333^ Considering the risk of hyperglycemia during infection, hospital protocols should include the management of hyperglycemia in COVID-19 patients.

Preexisting hyperglycemia or diabetes mellitus also worsens the situation of COVID-19 patients by affecting immune responses. Diabetes mellitus is characterized as a chronic inflammatory status. It is well-known that diabetes mellitus affects both innate and adaptive immunity.^334^ A previous clinical study shows that subjects with impaired glucose tolerance have higher levels of plasma inflammatory cytokines including IL-6, TNF-α, and IL-18 via an oxidative mechanism.^335^ Higher glucose levels stimulate inflammatory cytokine secretion of periphery blood mononuclear cells (PBMCs) and impede innate immunity by inhibiting type I IFN production.^336^ Another report indicates that COVID-19 patients with diabetes mellitus have higher levels of plasma inflammatory biomarkers including C-reactive protein, serum ferritin, IL-6, and a higher erythrocyte sedimentation rate.^337^ A retrospective multicenter study demonstrates that COVID-19 patients with diabetes have a higher likelihood of developing lymphopenia.^50^ Monocytes infected by SARS-CoV-2 produce more inflammatory cytokines, which are further augmented by higher glucose levels. Blockade of glycolysis by 2-DG significantly decreased the viral load and inflammatory cytokine secretion. From a mechanistic view, the infection induces ROS production to stabilize HIF-1α, consequently promoting glycolysis. This metabolic alteration of monocytes directly inhibits T cell responses, partially explaining the lymphopenia.^209^ Thus, hyperglycemia and diabetes mellitus likely trigger or exacerbate inflammation to culminate in poor prognosis of COVID-19 patients. The impaired innate immunity caused by high glucose levels is also conducive to SARS-CoV-2 infection.

***Lactate*** is a byproduct of glucose metabolism and the end-product of anaerobic glycolysis. Since SARS-CoV-2 infection enhances glycolysis, the level of lactate concomitantly increases. Recently, lactate is revealed to have diverse functions in regulating T cell proliferation, immune cell metabolism, macrophage polarization, and cytokine production.^338–341^ COVID-19 patients with higher lactate levels tend to worse outcomes.^342^ The lactate generation is catalyzed by lactate dehydrogenase (LDH). LDH regulates the interconversion of pyruvate and lactate, which is a crucial step in the anaerobic metabolism of glucose. The high LDH levels correlate with disease progression in COVID-19 patients.^328,343,344^ Besides being used as an indicator of severe COVID-19, lactate potentially contributes to disease progression. Lactate can rewire CD4+ T cell metabolism, leading to a deterioration of chronic inflammation diseases.^340^ Results from a previous study show that high lactate levels are associated with more prothrombotic fibrin properties and neutrophil extracellular trap (NET) formation, which are crucial mediators of coagulopathy in COVID-19.^345,346^ Moreover, lactate is an activator of HIF-1α. Lactate preconditioning can shift cellular glucose metabolism to glycolysis. By stabilizing HIF-1α, lactate promotes glycolysis in a positive feedback manner, thus facilitating SARS-CoV-2 infection.^347,348^ Intriguingly, lactate is also a suppressor of RLR-mediated innate immunity. Pattern recognition receptor RLRs are RNA sensors for triggering innate immune responses against viral infection. Lactate inhibits RLR signaling and type I IFN production by preventing MAVS mitochondrial localization/aggregation, and RIG-1-MAVS association. Reduction of lactate levels by pharmacological inhibition of lactate dehydrogenases A (LDHA) profoundly increased the resistance of mice against viral infection by upregulating type I IFN production.^349^ This finding explains the potential benefits of shifting glucose metabolism towards glycolysis for evading innate immune responses. However, the exact role of lactate in the pathogenesis of COVID-19 is intricate and not exhaustively studied.

Human intestines are inhabited by over 2000 species of microbes *(microbiota)*. It has been shown gut microbiota have many biological functions including shaping and modulating immune responses. Dysbiosis of gut microbiota could lead to aberrant immune responses or autoimmune diseases.^350,351^ More importantly, the composition of gut microbiota is closely related to inflammatory cytokine secretion, which is surprisingly reminiscent of the inflammatory comorbidities and cytokine storm caused by COVID-19.^352^ Analysis of stool samples from COVID-19 patients revealed that the composition of microbiota in COVID-19 patients underwent significant alterations. The abundance of probiotics was depleted meanwhile the abundance of opportunistic pathogens increased. The baseline abundance of *Coprobacillus*, *Clostridium ramosum*, and *Clostridium hathewayi* increases with disease severity. On the other hand, the abundance of *Faecalibacterium prausnitzii* inversely correlates with COVID-19 severity.^353^ Another study showed that COVID-19 patients had markedly less bacterial diversity, and the impaired bacterial diversity was associated with increased severity. This study also identified a decreased abundance of *Bifidobacterium*, *Faecalibacterium*, and *Roseburium*, and an increased abundance of *Bacteroides* in COVID-19 patients comparing to exposed controls.^354^ The levels of plasma cytokines and inflammatory markers in COVID-19 patients were closely associated with microbiota composition, linking gut microbiota to COVID-19-induced inflammatory disorders and tissue damage. Several species known to play immunoregulatory roles in human gastrointestinal tracts including *Bifidobacterium adolescentis*, *Eubacterium rectale*, and *Faecalibacterium prausnitzii* are depleted in COVID-19 patients. However, this study demonstrated no differences in bacterial diversity between COVID-19 patients and healthy controls.^355^ Since the study of human microbiota is still in its cradle, how SARS-CoV-2 affects microbiota is not fully elucidated yet. COVID-19 is accompanied by a set of sequelae even after complete disease resolution, namely long COVID-19 or post-COVID-19 syndromes, including fatigue, memory loss, anxiety, anosmia, and depression.^356,357^ Gut microbiota is closely related to long COVID-19. Altered microbiota composition and decreased bacterial diversity were detected in post-COVID-19 patients, even eight months after acute disease resolution.^358^ Patients with long COVID-19 had distinct microbiota composition compared to completely recovered patients. While there was no significant difference in microbiota between patients with or without antibiotic treatment, indicating that the difference was not due to antibiotic use. Intriguingly, different post-COVID-19 syndromes were associated with different microbiota patterns. For example, respiratory symptoms were positively correlated with opportunistic pathogens, while neuropsychiatric symptoms and fatigue were accompanied by changes in nosocomial pathogens. More importantly, composition at admission can predict the onset of post-COVID-19 syndromes, underlining the important role of microbiota.^359^ Of note, microbiota composition could be modified by dietary supplements. Some researchers suggested replenishment of probiotics by a plant-based rich fiber diet or other nutritional modulations that could be beneficial to COVID-19 patients.^360–363^ Otherwise, the influence on microbiota should be taken into consideration when using antibiotics for the treatment of COVID-19 patients. Compared with gut microbiota, lung microbiota was less investigated. However, recent findings support that lung microbiota also played an important role in COVID-19 pathology. Lung microbiota is involved in respiratory infections diseases and pneumonia.^364,365^ But due to the difficulty in measuring the composition of the microbial community in patients, the role of lung microbiota in COVID-19 is poorly understood.

As mentioned above, *long COVID-19* is manifested by a series of post-infection syndromes even a long period after the nucleic acid test shows a negative result. Over 200 symptoms have been identified in long COVID-19, some of them were severe and debilitating. For example, a meta-analysis showed that 22% of patients had cognitive impairment 12 weeks post-infection.^366^ Cognitive impairment is a canonical symptom of long COVID-19. The magnitude of cognitive impairment in long COVID-19 is equal to 10 years of cognitive aging, and likely increases over time.^367^ It is even more concerning that hospitalized and non-hospitalized patients have similar rates of developing cognitive impairment.^368^ Otherwise, many patients have other sequelae including multi-organ dysfunction, gastrointestinal disorders, respiratory disturbances, and cardiovascular disorders.^369–371^ The etiology of long COVID-19 is multifactorial. Major contributors to long COVID-19 are proposed to be abnormally altered immune system, circular system dysfunction, multi-organ damage, and neuroinflammation.^372,373^ Considering the deleterious effect of long COVID-19, exploring the mechanism and prophylaxis is of importance. Many long COVID-19 patients shared similar symptoms with Myalgic encephalomyelitis/chronic fatigue syndromes (ME/CFS), a multisystem neuroimmune disorder that generally happens after infection. ME/CFS was manifested by chronic fatigue, cognitive impairment, and post-exertional malaise.^374,375^ Therefore, therapeutic strategy for ME/CFS presumably also alleviated COVID-19. Dietary supplementation of coenzyme Q can reduce fatigue and oxidative stress in ME/CFS.^376^ The effect of coenzyme Q on long COVID-19 is being clinically tested. It is also noted that mast cell activation was prevalent in long COVID-19 patients, implying the causal link between mast cell activation and long COVID-19.^377,378^ The proposed mechanism is that aberrant mast cell activation triggers mediators including histamine, cytokines, leukotrienes, and prostaglandins, bringing damage to multiple tissues. Antihistamine treatment is the protocol for mast cell activation syndrome. Pilot studies have demonstrated that antihistamines can relieve long COVID-19-associated symptoms.^379,380^ Some components of traditional herbs including quercetin and luteolin are also proposed for the prevention or treatment of long COVID-19, although there is no clinical evidence till now.^381^ It is noteworthy that physical exercises exacerbate the condition of COVID-19 patients instead of improving it. According to a survey, regular physical exercises worsened the condition of 74.84% long COVID-19 patients, only 0.84% patients reported improvement by physical exercises.^382^ Hence, long COVID-19 patients should take caution when practicing. Although long COVID-19 brings enormous health burdens to patients, there are few clinical studies available now.

### Drug repurposing

Patients with severe COVID-19 undergoing mechanical ventilation have a poor prognosis, and even some recovered patients have severe sequelae, such as lung fibrosis, chronic vasculitis, hypertension, and embolism.^383,384^ Considering the urgency, repurposing FDA-proved drugs with a verified safety profile is of interest. This section focuses on the repurposing of FDA-proved drugs that intervene in host lipid metabolism for the treatment of COVID-19 and their clinical research. The clinical trials are summarized in Table 1.

**Table 1:** Summary of metabolism-modulating drugs

Drug name	Clinical trial	Phase
Statins		
Simvastatin	NCT04348695	II
	NCT05542095	I
Rosuvastatin	NCT04472611	III
	NCT04359095	III
	NCT04472611	III
	NCT05594615	I
Atorvastatin	NCT04631536	III
	NCT04486508	III
	NCT04952350	III
	NCT04380402	II
	NCT04813471	III
	NCT04904536	III
	NCT04801940	III
	NCT04380402	II
	NCT04466241	III
ASM inhibitors		
Fluoxetine	NCT04377308	IV
	NCT05041907	II
	NCT05283954	III
	NCT04920838	III
	NCT04780152	III
Fluvoxamine	NCT04718480	II
	NCT04342663	II
	NCT04668950	III
	NCT04727424	III
	NCT05087381	IV
	NCT04510194	III
	NCT04711863	II
NSAIDs		
Aspirin	NCT05073718	III
	NCT04368377	II
	NCT04324463	III
	NCT04365309	III
	NCT04381936	III
	NCT04808895	III
	NCT04466670	II
	NCT04937088	II
	NCT04768179	III
Paracetamol	NCT04920838	III
	NCT04673214	III
	NCT04416334	III
	NCT04536051	III
Naproxen	NCT04325633	III
Montelukast		
Montelukast	NCT04718285	II
	NCT04695704	III
	NCT05094596	IV
	NCT04389411	III
Omega-3 fatty acids		
Omega-3 fatty acids	NCT04836052	III
	NCT05121766	I
	NCT04647604	II
	NCT04495816	II
	NCT04553705	III
	NCT04460651	III
	NCT04335032	III
	NCT05711810	IV
2-DG		
No available clinical trials		
Metformin		
Metformin Metformin glycinate	NCT04510194 NCT04626089 NCT04625985	III II II

#### Statins

Statins have been applied in clinic for decades with a proven efficacy and safety for the treatment of hyperlipidemia and as prophylaxis of atherosclerosis disease.^385^ HMG-CoA (3-hydroxy-3-methyl-glutaryl-CoA) reductase (HMGCR) is a rate-limiting enzyme for cholesterol biosynthesis. Statins are strong competitive inhibitors of HMGCR and are generally used as lipid-lowering drugs. Statins effectively reduce plasma cholesterol levels by inhibiting cholesterol biosynthesis and lowering low-density lipoprotein (LDL) levels.^386^ Since cellular cholesterol plays a critical role in SARS-CoV-2 viral entry, statins have bright prospects for treating COVID-19.

Clinical reports of COVID-19 patients revealed a correlation between cholesterol metabolism and prognosis. Statistical analysis shows that a higher serum high-density lipoprotein cholesterol (HDLc) level predicts a lower mortality rate before infection by SARS-CoV-2.^310^ Total cholesterol, LDL, and HDL levels decreased in COVID-19 patients compared to the levels prior to infection. Total cholesterol and LDL recovered to baseline in discharged patients but progressively dropped in non-survival patients.^387^ Given that statins manage cholesterol profiles, they could improve the conditions of COVID-19 patients.

Aside from regulating cholesterol levels, statins have diverse effects independent of HMGCR inhibition, including stablizing endothelial dysfunction, regulating atherosclerosis, anti-fibrosis, anti-thrombosis, anti-oxidantion, anti-apoptosis, and anti-inflammation.^388^ The impact of statins on ARDS is controversial, two clinical studies show no improvement in ARDS under statins treatment versus placebo.^389,390^ While another study indicates a potential benefit to ARDS patients associated with statin use.^391^ Meta-analysis indicates that statin practically prolongs ventilator-free days of patients with ARDS, although it does not improve mortality and severe sepsis.^392^

A retrospective study of COVID-19 patients shows that the 28-day all-cause mortality rate was 5.2% and 9.4% in the matched statin and non-statin groups, respectively.^393^ Another retrospective study confirms that COVID-19 patients admitted to ICU who used atorvastatin had a slower progression to death and a lower mortality rate.^394^ Meta-analysis of 19 studies involving over 395,000 patients indicates that prior statin use is associated with a lower risk of mortality and a reduced risk of severe COVID-19.^395^ However, another study obtained a different result: prior treatment of statins improves neither severity outcome nor mortality rates. The contradictory results may be caused by the risk of exacerbating compensatory immune signals through the effect of statins on TLR and NF-κB.^396^ In addition, results from clinical studies show that statin treatment upregulates ACE2 expression, thereby potentially facilitating SARS-CoV-2 attachment and entry. Hence, whether statins practically improve COVID-19 severity and prognosis or not remains a matter of debate.^397^

#### ASM inhibitors

Most antidepressants belong to functional inhibitors of acid sphingomyelinase (FIASMAs) and are widely applied as psychotropic medications. Antidepressants, such as fluoxetine, fluvoxamine, paroxetine, and amitriptyline, have been widely used for decades with proven safety.^398^ As discussed above, evidence supports the central role of ASM/ceramide system during SARS-CoV-2 infection. FIASMAs, which practically reduce ceramide levels, are promising to serve as a therapeutic modality for COVID-19. They regulate pro-inflammatory cytokine secretion and thereby display anti-inflammatory properties. They also interact with ER chaperone the Sigma-1 receptor and ER stress sensor inositol-requiring enzyme 1α to regulate cytokine secretion and immune response.^399^ Besides blocking SARS-CoV-2 viral entry, FIASMAs can also ameliorate COVID-19 via their immune modulatory property.^400,401^

The potency of FIASMAs has been validated. A multicenter study on hospitalized severe COVID-19 patients shows a reduced risk of intubation or death under FIASMA treatment (37.5% FIASMA group vs 41.4% non-FIASMA group). The correlation between taking FIASMAs and reduced likelihood of intubation or death is not specific to one particular class of FIASMAs.^402^ Another cohort study on severe COVID-19 patients reveals that chronic prescription of FIASMA is associated with a lower risk of mortality. In this study, amlodipine exhibited the most prominent efficacy among FIASMAs. Patients treated with amlodipine before the infection had a lower rate of mortality (12.7%) than patients not treated with amlodipine before the infection (34.9%).^403^ In a randomized double-blinded clinical trial, fluvoxamine reduced the likelihood of clinical deterioration compared to the placebo group.^404^ In another randomized clinical trial, the proportion of patients observed in a COVID-19 emergency setting or transferred to a tertiary hospital due to COVID-19 was lower in the fluvoxamine group compared with the placebo group.^405^ Collectively, these clinical results support FIASMAs as an effective modality for COVID-19.

#### NSAIDs

Non-steroidal anti-inflammatory drugs (NSAIDs), the COX-1 and COX-2 inhibitors, are prospective as a therapy for COVID-19. NSAIDs are well-tolerated anti-inflammatory drugs that are widely used for managing acute or chronic inflammatory diseases. A retrospective study indicates that the use of NSAIDs is associated with a reduced risk of hospitalization of COVID-19 patients with chronic inflammatory diseases.^406^ Another study obtained a similar conclusion: COVID-19 patients prescribed ibuprofen or naproxen had a lower risk of hospitalization.^407^

However, the application of NSAIDs on COVID-19 remains controversial. Some propose that NSAIDs usage upregulates ACE2 expression, thus promoting SARS-CoV-2 entry while other studies demonstrate that NSAIDs have no impact on ACE2 expression and will not exacerbate infection.^408–411^ There are also concerns that NSAIDs could exacerbate hypercoagulation and the incidence of thrombosis due to decreased thrombomodulin caused by NSAID treatment.^412,413^ Furthermore, NSAIDs may predispose patients to gastrointestinal and cardiovascular complications which are also canonical symptoms of COVID-19.^414^

Although the World Health Organization has declared that there is no evidence of an increased risk of disease deterioration, considering the potential adverse effects, NSAIDs should be used with caution on COVID-19 patients.

#### Montelukast

Montelukast is a cysteinyl leukotriene receptor antagonist that blocks the binding of cysteinyl leukotrienes. Montelukast was initially used for the treatment of chronic asthma with excellent safety. Montelukast can effectively suppress inflammation by inhibiting NF-κB activation and the downstream pro-inflammatory cytokines (IL-6, TNF-α, MCP-1, et al.) secretion in cultured human mononuclear cells and macrophages upon stimulation.^415,416^ In addition, montelukast is also capable of inhibiting platelet activation under the stimulation of serum from COVID-19 patients by downregulating the surface expression of tissue factors and P-selectin. As a consequence, the formation of monocyte- and granulocyte-platelet aggregates are profoundly inhibited.^417^

Montelukast also affects the viral proteins of SARS-CoV-2. A computational simulation predicts that montelukast may bind S protein to disturb the RBD domain.^418^ Montelukast sodium hydrate binds with the C-terminus domain of SARS-CoV-2 NSP1 protein to restore host protein synthesis, which is suppressed by NSP1.^419,420^

Hospitalized patients treated with montelukast experienced significantly fewer events of clinical deterioration compared with patients not receiving montelukast (10% vs 32%).^421^ Montelukast treatment alleviated the severity of COVID-19, prevented lung respiratory failure, and reduced mortality.^422^ Montelukast is currently under phase III clinical trial (NCT04389411) for the treatment of severe COVID-19.

#### Omega-3 fatty acids

Pro-resolving mediators, e.g., lipoxins, resolvins, protectins and maresins, stimulate key cellular events in resolution, namely cessation of neutrophil infiltration and enhanced macrophage uptake of debris, hence actively protecting tissues from hyperinflammation and expediting recovery from inflammatory damage.^423^ Treatment with such pro-resolving mediators accelerates the clearance of bacteria and impedes neutrophil accumulation in the lung, thereby promoting the resolution of bacteria-induced lung injury.^275^ These pro-resolving mediators are synthesized from two Omega-3 fatty acids: eicosapentaenoic acid (EPA) and docosahexaenoic (DHA). Both serve as precursors in the biochemical pathways leading to Specialized pro-resolving mediators.^424^ Therefore, exogenous supplementation of omega-3 fatty acids is recommended to treat COVID-19 via the pro-resolving process.^425^

Omega-3 index (O3I) reflects blood levels of EPA and DHA. It was revealed a lower O3I as a risk factor for severe COVID-19A study after comparison of O3I levels in hospitalized severe COVID-19 patients with ambulatory patients with mild infection.^426^ A randomized double-blinded study was conducted to investigate the therapeutic effect of omega-3 fatty acid supplementation on severe COVID-19 patients. The result is encouraging, it showed that omega-3 fatty acid supplementation improved the levels of several parameters of respiratory and renal function in critically ill patients and increased the survival rate.^427^ Omega-3 fatty acids are well-known as anti-inflammatory and pro-resolving factors and confirmed to improve the conditions of COVID-19 patients.^425,428^

#### 2-DG

2-DG is a synthetic analog of glucose that interferes with glycolysis. 2-DG has been used in diverse areas for decades, including antivirus, anticancer, and antiepileptic.^429–431^ As mentioned above, SARS-CoV-2 induces the Warburg effect, and its infection highly depends on glycolysis. The transition of glucose metabolism to glycolysis favors SARS-CoV-2 entry, replication, and pathogenesis. Moreover, 2-DG also inhibits the PPP pathway and glycosylation of S protein. Hence, 2-DG is presumably promising to ameliorate the severity of COVID-19.^202,203^

2-DG treatment significantly reduced the viral load of cells infected by SARS-CoV-2 and ameliorated the CPE and cell death. Surprisingly, the progeny SRAS-CoV-2 generated from 2-DG-treated cells exhibited weakened infectivity.^432^ However, 2-DG has potential toxicity. 2-DG can cause glucocytopenia in the nervous system, and irregularities in the cardiovascular, respiratory, and immunological systems.^433,434^ In a randomized phase II clinical study, 2-DG was administered as an adjunct to stand of care (SOC). Patients treated with 90 mg/kg/day 2-DG had better outcomes. The clinical recovery and vital signs normalization were faster in the 90 mg/kg/day 2-DG group. However, in this study, 30.3% of patients reported adverse events.^435^ In 2021, 2-DG was approved by the Drug Controller General of India as an adjunct therapy along with the SOC in hospitalized patients with mild to severe COVID-19. The clinical data indicates that patients treated with 2-DG + SOC underwent a quicker symptomatic relief and normalization of vital sign factors compared with SOC alone. The clinical data is accessible from the trials’ registrations on the Clinical Trial Registry of India. Notably, continuous administrations of high 2-DG doses over a long time might cause toxicity to the individuals. Considering the adverse effects, 2-DG should be used with caution.

#### Metformin

Metformin has been used as a first-line anti-diabetic drug for decades. Recent research reveals that metformin also possesses anti-cancer, anti-aging, anti-inflammatory, and other properties.^436–439^ Due to the prominent bioactivity of metformin, it is also proposed for the treatment of COVID-19.

Several observational studies have confirmed the beneficial effect of metformin on COVID-19. As mentioned above, diabetic mellitus, dysregulated glucose metabolism, and hyperglycemia are risk factors for severe COVID-19. A large-scale retrospective study including over 2,800,000 COVID-19 patients with diabetes has been conducted to evaluate the influence of different glucose-lowering drugs. Patients prescribed metformin have markedly less COVID-19-related mortality rates compared with those prescribed insulin. However, several confounding factors have not been adjusted in this study.^440^ Another two small-scale retrospective studies indicate that, after adjusting confounding factors, metformin use is still associated with lower mortality rates in COVID-19 patients with diabetes.^441,442^ Intriguingly, the beneficial effect of metformin is shown to be gender-dependent. Metformin treatment profoundly alleviates the severity in female COVID-19 patients, while the effect on male patients is minimal. Researchers propose that this difference is probably due to the stronger suppressive effect of metformin on TNF-α production in women than in men, suggesting that metformin alleviates COVID-19 primarily via regulating inflammatory cytokines.^443^ Several clinical trials are currently being conducted. Those clinical studies demonstrate that metformin treatment prevented disease progression and reduced mortality rates. However, further studies are needed through randomized, double-blinded clinical trials.

From a mechanistic perspective, metformin presumably ameliorates COVID-19 via multifaceted effects. Metformin can prevent endothelial dysfunction, reduce cytokine release, improve glucose metabolism, and promote resolution of lung damage after acute inflammation. All these effects are considered to be responsible for alleviating COVID-19 severity.^444–448^ Considering the distinct therapeutic effect between males and females, the anti-inflammatory effect seems to play a major role. However, the practical mechanism needs to be experimentally investigated.

## Summary and perspective

In this review, we summarize the advances and provide comprehensive knowledge of metabolic alterations induced by SARS-CoV-2 and how they influence SARS-CoV-2 infection in aspects of entry, replication, assembly, and pathogenesis. We also include studies of drugs repurposed for COVID-19.

Like other coronaviruses, SARS-CoV-2 entry entails the intact lipid rafts on cell membranes.^449–451^ The paramount importance of lipid rafts during SARS-CoV-2 entry was underpinned and summarized by several researchers.^75,452^ The role of cellular cholesterol metabolism is controversial and not fully interpreted. Some researchers suggest that SARS-CoV-2 downregulates cellular cholesterol levels, and increased cholesterol levels impede SARS-CoV-2 infection. However, others suggest that cholesterol depletion restrains SARS-CoV-2 infection. The inconsistent results could be due to different time points, positions, treatments, methods, and so forth. The exact role of cholesterol entails further studies. Cholesterol might influence SARS-CoV-2 infection in post-entry stages including replication, virion maturation, and egress, although there is currently no available study. It would be also interesting to interrogate how increased cellular cholesterol restrains SARS-CoV-2 infection. Ceramides assist SARS-CoV-2 entry by forming ceramide-rich microdomains where ACE2 clusters. Contrarily, sphingosine, the downstream metabolite of ceramides, impedes SARS-CoV-2 entry by blocking the interaction between S protein and ACE2. S1P is a product downstream of sphingosine. The anti-inflammatory and anti-thrombotic properties of S1P are well-established. S1P is postulated to ameliorate COVID-19 by protecting endothelial barrier, although there is no available study.^453,454^ Considering the intimate relationship between COVID-19 and sphingomyelin metabolism, other components in sphingomyelin are probably involved in different stages of SARS-CoV-2 infection. Interrogating the ER branch of sphingomyelin metabolism would be of interest since SARS-CoV-2 replication organelles are also derived from ER. Lipid modifications of S protein have been intensively investigated. The modification by linoleic acid locks the S protein in a closed conformation and prevents viral entry, while the palmitoylation of the S protein stabilizes the S protein homotrimer, thereby promoting viral entry. S protein of MHV is also demonstrated to be palmitoylated, and mutation at the palmitoylation sites impeded MHV penetration into cells and reduced specific infectivity, indicating the conservation of S protein palmitoylation among coronaviruses.^455^ Hyperglycemia or diabetes mellitus facilitates SARS-CoV-2 entry by upregulating ACE2, partially accounting for the worse situation of COVID-19 patients with preexisting diabetes. The glycosylation of S protein also significantly contributes to viral entry.

Host lipids also contribute to SARS-CoV-2 replication. The glycerophospholipid metabolism pathway facilitates SARS-CoV-2 replication by facilitating DMV formation. However, which step of the glycerophospholipid metabolism is responsible remains unknown. PE and PC are downstream metabolites of PA, and both were reported to hijack RNA virus replication,^173,175,176,456^ but currently there is no available data on the role of PE and PC in SARS-CoV-2 replication. Deciphering the roles of PC and PE during SARS-CoV-2 infection is of interest. The employment of host LDs by SARS-CoV-2 to meet the energy and material demand for replication was evidenced by current findings. The involvement of LDs was well-elucidated in other RNA viruses including hepatitis C virus, rotavirus, Zika virus, and dengue virus.^181,457–460^ The PPP generates nucleotides for viral replication, partially explaining the benefit of the Warburg effect induced by SARS-CoV-2. The onset of the Warburg effect is also seen in other virus infection.^205,461,462^ One carbon metabolism provides material for SARS-CoV-2 RNA capping, which is important for viral replication. The cap formation is conveyed by NSPs, primarily NSP9, and NSP12.^463,464^ Capping at the 5’ of viral RNA can prevent RNA degradation by innate immune responses and facilitates viral protein translation.^222^ Of note, phosphatidylinositol phosphate biosynthesis is revealed to be essential for SARS-CoV-2 infection. SARS-CoV-2 infection is highly dependent on phosphatidylinositol phosphate biosynthesis.^86,87^ In addition to contributing to replication compartment formation, phosphatidylinositol phosphate biosynthesis likely promotes SARS-CoV-2 in different stages of its life cycle. Further studies would be of interest.

The next section depicted how host lipids assist SARS-CoV-2 assembly. M protein uses several lipid components to induce membrane curvature. E protein palmitoylation is supported by computational simulations, and the adduct of palmitate is able to maintain E protein functional architecture. It was previously hypothesized that β-coronaviruses employ the biosynthetic pathway for egress. However, recent findings suggested β-coronaviruses employ deacidified lysosomes for virion release.^28,465,466^ Due to a paucity of comprehension of coronavirus egress mechanism, related studies are insufficient. Considering that the lysosomes are intimately connected to the lipid metabolism of LDs and ER, research on the association between host lipids and viral exit is warranted.^467,468^

Severe COVID-19 is manifested by ARDS, hyperinflammatory status, thrombotic complications, and multi-organ failures. This paper reviewed the impact of host lipids on COVID-19 pathogenesis. Eicosanoids of the AA pathway including prostaglandins, thromboxanes, and leukotrienes as well as the ASM/ceramide system are included as they affect thrombotic and inflammatory status during SARS-CoV-2 infection. The importance of cholesterol in COVID-19 is interpreted in this section. The bidirectional relationship between COVID-19 and dysregulated glucose metabolism was discussed. The role of lactate is also incorporated. Recent studies indicate that many COVID-19 patients underwent microbiota dysbiosis, and microbiota associates with COVID-19 severity via the gut-lung axis.^469–471^ The alteration of microbiota composition and its important role in COVID-19 is exhaustively discussed. However, available studies on the mechanism underlying the onset of long COVID-19 are few. Treatments and prophylaxis for long COVID-19 needs to be further validated by clinical studies.

Ultimately, several lipid-modulating and glucose-modulating drugs repurposed for COVID-19 are overviewed. Statins, ASM inhibitors, NSAIDs, Montelukast, Omega-3 fatty acids, 2-DG, and metformin are summarized. Further long-term follow-up studies are needed to characterize the benefit of these drugs for treating COVID-19 patients. Coronaviruses have given rise to three pandemics with huge damage to our society in the last two decades. However, therapies with proven efficacy are still lacking till now. Understanding the interaction between coronaviruses and host metabolism will prompt antiviral drug development and better disease management that would apply not only to SARS-CoV-2 infection, but also potentially to other types of coronavirus infections.

## References

[1] He M-L (2006). Kinetics and synergistic effects of siRNAs targeting structural and replicase genes of SARS-associated coronavirus. Febs. Lett..

[2] de Wit E, van Doremalen N, Falzarano D, Munster VJ (2016). SARS and MERS: recent insights into emerging coronaviruses. Nat. Rev. Microbiol..

[3] Harrison AG, Lin T, Wang P (2020). Mechanisms of SARS-CoV-2 transmission and pathogenesis. Trends Immunol..

[4] Wu F (2020). A new coronavirus associated with human respiratory disease in China. Nature.

[5] Lu R (2020). Genomic characterisation and epidemiology of 2019 novel coronavirus: implications for virus origins and receptor binding. Lancet.

[6] Chen N (2020). Epidemiological and clinical characteristics of 99 cases of 2019 novel coronavirus pneumonia in Wuhan, China: a descriptive study. Lancet.

[7] Zhou F (2020). Clinical course and risk factors for mortality of adult inpatients with COVID-19 in Wuhan, China: a retrospective cohort study. Lancet.

[8] Chan W, He B, Wang X, He ML (2020). Pandemic COVID-19: current status and challenges of antiviral therapies. Genes Dis..

[9] Masters PS (2006). The molecular biology of Coronaviruses. Adv. Virus Res.

[10] Perlman S, Netland J (2009). Coronaviruses post-SARS: update on replication and pathogenesis. Nat. Rev. Microbiol..

[11] Zhou F (2021). Attenuating innate immunity and facilitating beta-coronavirus infection by NSP1 of SARS-CoV-2 through specific redistributing hnRNP A2/B1 cellular localization. Signal Transduct. Target. Ther..

[12] Yang H, Rao Z (2021). Structural biology of SARS-CoV-2 and implications for therapeutic development. Nat. Rev. Microbiol..

[13] Tortorici MA, Veesler D (2019). Structural insights into coronavirus entry. Adv. Virus Res..

[14] Ruch TR, Machamer CE (2012). The coronavirus E protein: assembly and beyond. Viruses.

[15] V’Kovski P, Kratzel A, Steiner S, Stalder H, Thiel V (2021). Coronavirus biology and replication: implications for SARS-CoV-2. Nat. Rev. Microbiol..

[16] Satarker S, Nampoothiri M (2020). Structural proteins in severe acute respiratory syndrome coronavirus-2. Arch. Med. Res..

[17] Scherer KM (2022). SARS-CoV-2 nucleocapsid protein adheres to replication organelles before viral assembly at the Golgi/ERGIC and lysosome-mediated egress. Sci. Adv..

[18] Lu X, Pan J, Tao J, Guo D (2011). SARS-CoV nucleocapsid protein antagonizes IFN-beta response by targeting initial step of IFN-beta induction pathway, and its C-terminal region is critical for the antagonism. Virus Genes.

[19] Li F (2016). Structure, function, and evolution of coronavirus spike proteins. Annu. Rev. Virol..

[20] Romero-Brey I, Bartenschlager R (2014). Membranous replication factories induced by plus-strand RNA viruses. Viruses.

[21] Miller S, Krijnse-Locker J (2008). Modification of intracellular membrane structures for virus replication. Nat. Rev. Microbiol..

[22] Cortese M (2020). Integrative imaging reveals SARS-CoV-2-induced reshaping of subcellular morphologies. Cell Host Microbe.

[23] Snijder EJ (2006). Ultrastructure and origin of membrane vesicles associated with the severe acute respiratory syndrome coronavirus replication complex. J. Virol..

[24] Wolff G, Melia CE, Snijder EJ, Barcena M (2020). Double-membrane vesicles as platforms for viral replication. Trends Microbiol.

[25] Wolff G (2020). A molecular pore spans the double membrane of the coronavirus replication organelle. Science.

[26] de Haan CAM, Rottier PJM (2005). Molecular interactions in the assembly of coronaviruses. Adv. Virus Res..

[27] Stertz S (2007). The intracellular sites of early replication and budding of SARS-coronavirus. Virology.

[28] Ghosh S (2020). beta-Coronaviruses use lysosomes for egress instead of the biosynthetic secretory pathway. Cell.

[29] Mendonca, L. et al. SARS-CoV-2 assembly and egress pathway revealed by correlative multi-modal multi-scale cryo-imaging. Preprint at *bioRxiv*10.1101/2020.11.05.370239 (2020).

[30] Strating JR, van Kuppeveld FJ (2017). Viral rewiring of cellular lipid metabolism to create membranous replication compartments. Curr. Opin. Cell Biol..

[31] Martin-Acebes MA, Vazquez-Calvo A, Saiz JC (2016). Lipids and flaviviruses, present and future perspectives for the control of dengue, Zika, and West Nile viruses. Prog. Lipid Res..

[32] Blaising J, Pecheur EI (2013). Lipids: a key for hepatitis C virus entry and a potential target for antiviral strategies. Biochimie.

[33] Chan RB, Tanner L, Wenk MR (2010). Implications for lipids during replication of enveloped viruses. Chem. Phys. Lipids.

[34] Altan-Bonnet N (2017). Lipid tales of viral replication and transmission. Trends Cell Biol..

[35] Monson EA, Trenerry AM, Laws JL, Mackenzie JM, Helbig KJ (2021). Lipid droplets and lipid mediators in viral infection and immunity. Fems. Microbiol. Rev..

[36] Monson EA, Whelan DR, Helbig KJ (2021). Lipid droplet motility increases following viral immune stimulation. Int. J. Mol. Sci..

[37] Grootemaat AE (2022). Lipid and nucleocapsid N-protein accumulation in COVID-19 patient. Microbiol. Spectr..

[38] Nardacci R (2021). Evidences for lipid involvement in SARS-CoV-2 cytopathogenesis. Cell Death Dis..

[39] Song JW (2020). Omics-driven systems interrogation of metabolic dysregulation in COVID-19 pathogenesis. Cell Metab..

[40] Wu D (2020). Plasma metabolomic and lipidomic alterations associated with COVID-19. Natl Sci. Rev..

[41] Lunt SY, Vander Heiden MG (2011). Aerobic glycolysis: meeting the metabolic requirements of cell proliferation. Annu. Rev. Cell. Dev. Biol..

[42] Zhu L, Zhao Q, Yang T, Ding W, Zhao Y (2015). Cellular metabolism and macrophage functional polarization. Int. Rev. Immunol..

[43] Lee AH, Dixit VD (2020). Dietary regulation of immunity. Immunity.

[44] Kang S, Tang H (2020). HIV-1 infection and glucose metabolism reprogramming of T cells: another approach toward functional cure and reservoir eradication. Front. Immunol..

[45] Van den Bossche J, O’Neill LA, Menon D (2017). Macrophage immunometabolism: where are we going?. Trends Immunol..

[46] Willig AL, Overton ET (2016). Metabolic complications and glucose metabolism in HIV infection: a review of the evidence. Curr. HIV/AIDS Rep..

[47] Vander Heiden MG, Cantley LC, Thompson CB (2009). Understanding the Warburg effect: the metabolic requirements of cell proliferation. Science.

[48] Lim S, Bae JH, Kwon HS, Nauck MA (2021). COVID-19 and diabetes mellitus: from pathophysiology to clinical management. Nat. Rev. Endocrinol..

[49] Williamson EJ (2020). Factors associated with COVID-19-related death using OpenSAFELY. Nature.

[50] Zhu L (2020). Association of blood glucose control and outcomes in patients with COVID-19 and pre-existing type 2 diabetes. Cell metab..

[51] Zhao Q (2022). Metabolic modeling of single bronchoalveolar macrophages reveals regulators of hyperinflammation in COVID-19. iScience.

[52] Harding JL (2022). The bidirectional association between diabetes and long-COVID-19 - A systematic review. Diabetes Res. Clin. Pract..

[53] Rey-Renones C (2022). Type 2 diabetes mellitus and COVID-19: a narrative review. Biomedicines.

[54] Gusev E, Sarapultsev A, Solomatina L, Chereshnev V (2022). SARS-CoV-2-specific immune response and the pathogenesis of COVID-19. Int. J. Mol. Sci..

[55] Amraei R (2021). CD209L/L-SIGN and CD209/DC-SIGN Act as Receptors for SARS-CoV-2. Acs. Cent. Sci..

[56] Wang S (2021). AXL is a candidate receptor for SARS-CoV-2 that promotes infection of pulmonary and bronchial epithelial cells. Cell Res..

[57] Koch J (2021). TMPRSS2 expression dictates the entry route used by SARS-CoV-2 to infect host cells. EMBO J..

[58] Hoffmann M (2020). SARS-CoV-2 cell entry depends on ACE2 and TMPRSS2 and is blocked by a clinically proven protease inhibitor. Cell.

[59] Beumer J (2021). A CRISPR/Cas9 genetically engineered organoid biobank reveals essential host factors for coronaviruses. Nat. Commun..

[60] Kuhn JH, Li W, Choe H, Farzan M (2004). Angiotensin-converting enzyme 2: a functional receptor for SARS coronavirus. Cell Mol. Life. Sci..

[61] Zhou T (2020). Cryo-EM structures of SARS-CoV-2 spike without and with ACE2 reveal a pH-dependent switch to mediate endosomal positioning of receptor-binding domains. Cell Host Microbe.

[62] Wei J (2021). Genome-wide CRISPR screens reveal host factors critical for SARS-CoV-2 infection. Cell.

[63] Bayati A, Kumar R, Francis V, McPherson PS (2021). SARS-CoV-2 infects cells after viral entry via clathrin-mediated endocytosis. J. Biol. Chem..

[64] Jackson CB, Farzan M, Chen B, Choe H (2022). Mechanisms of SARS-CoV-2 entry into cells. Nat. Rev. Mol. Cell Biol..

[65] Simons K, Sampaio JL (2011). Membrane organization and lipid rafts. Cold Spring Harb. Perspect. Biol..

[66] Sorice M (2020). Targeting lipid rafts as a strategy against coronavirus. Front. Cell. Dev. Biol..

[67] Fecchi K (2020). Coronavirus interplay with lipid rafts and autophagy unveils promising therapeutic targets. Front. Microbiol..

[68] Peruzzu D (2022). Zika virus exploits lipid rafts to infect host cells. Viruses.

[69] Palacios-Rapalo SN (2021). Cholesterol-rich lipid rafts as platforms for SARS-CoV-2 entry. Front. Immunol..

[70] Glende J (2008). Importance of cholesterol-rich membrane microdomains in the interaction of the S protein of SARS-coronavirus with the cellular receptor angiotensin-converting enzyme 2. Virology.

[71] Lu Y, Liu DX, Tam JP (2008). Lipid rafts are involved in SARS-CoV entry into Vero E6 cells. Biochem. Biophys. Res. Commun..

[72] Warner FJ (2005). Angiotensin-converting enzyme 2 (ACE2), but not ACE, is preferentially localized to the apical surface of polarized kidney cells. J. Biol. Chem..

[73] Sim JR (2022). Amelioration of SARS-CoV-2 infection by ANO6 phospholipid scramblase inhibition. Cell Rep..

[74] George KS, Wu S (2012). Lipid raft: a floating island of death or survival. Toxicol. Appl. Pharmacol..

[75] Li X (2021). Dependence of SARS-CoV-2 infection on cholesterol-rich lipid raft and endosomal acidification. Comput. Struct. Biotechnol. J..

[76] Wang S (2020). Cholesterol 25-Hydroxylase inhibits SARS-CoV-2 and other coronaviruses by depleting membrane cholesterol. EMBO J..

[77] Compton AA, Schwartz O (2017). They might be giants: does syncytium formation sink or spread HIV infection?. PLoS. Pathog..

[78] Duelli D, Lazebnik Y (2007). Cell-to-cell fusion as a link between viruses and Cancer. Nat. Rev. Cancer.

[79] Buchrieser J (2020). Syncytia formation by SARS-CoV-2-infected cells. EMBO J..

[80] Sanders DW (2021). SARS-CoV-2 requires cholesterol for viral entry and pathological syncytia formation. eLife.

[81] Musarrat F (2020). The anti-HIV drug nelfinavir mesylate (Viracept) is a potent inhibitor of cell fusion caused by the SARSCoV-2 spike (S) glycoprotein warranting further evaluation as an antiviral against COVID-19 infections. J. Med. Virol..

[82] Niyogi K, Hildreth JE (2001). Characterization of new syncytium-inhibiting monoclonal antibodies implicates lipid rafts in human T-cell leukemia virus type 1 syncytium formation. J. Virol..

[83] Wei C (2020). HDL-scavenger receptor B type 1 facilitates SARS-CoV-2 entry. Nat. Metab..

[84] Shen WJ, Asthana S, Kraemer FB, Azhar S (2018). Scavenger receptor B type 1: expression, molecular regulation, and cholesterol transport function. J. Lipid Res..

[85] Correa Y (2021). SARS-CoV-2 spike protein removes lipids from model membranes and interferes with the capacity of high density lipoprotein to exchange lipids. J. Colloid Interface Sci..

[86] Wang R (2021). Genetic screens identify host factors for SARS-CoV-2 and common cold coronaviruses. Cell.

[87] Daniloski Z (2021). Identification of required host factors for SARS-CoV-2 infection in human. Cells Cell.

[88] Campanero-Rhodes MA (2007). N-glycolyl GM1 ganglioside as a receptor for simian virus 40. J. Virol..

[89] Miller ME, Adhikary S, Kolokoltsov AA, Davey RA (2012). Ebolavirus requires acid sphingomyelinase activity and plasma membrane sphingomyelin for infection. J. Virol..

[90] Hayashi Y (2014). Sphingomyelin synthase 2, but not sphingomyelin synthase 1, is involved in HIV-1 envelope-mediated membrane fusion. J. Biol. Chem..

[91] Aizaki H (2008). Critical role of virion-associated cholesterol and sphingolipid in hepatitis C virus infection. J. Virol..

[92] Hannun YA, Obeid LM (2018). Sphingolipids and their metabolism in physiology and disease. Nat. Rev. Mol. Cell Biol..

[93] Vitner EB, Avraham R, Politi B, Melamed S, Israely T (2021). Elevation in sphingolipid upon SARS-CoV-2 infection: possible implications for COVID-19 pathology. Life Sci. Alliance.

[94] Torretta TE (2021). Severity of COVID-19 patients predicted by serum sphingolipids signature. Int. J. Mol. Sci..

[95] Carpinteiro A (2020). Pharmacological inhibition of acid sphingomyelinase prevents uptake of SARS-CoV-2 by epithelial cells. Cell Rep. Med..

[96] Grassme H (2003). Host defense against *Pseudomonas aeruginosa* requires ceramide-rich membrane rafts. Nat. Med..

[97] Grassme H (2001). CD95 signaling via ceramide-rich membrane rafts. J. Biol. Chem..

[98] Carpinteiro A (2021). Inhibition of acid sphingomyelinase by ambroxol prevents SARS-CoV-2 entry into epithelial cells. J. Biol. Chem..

[99] Schloer S (2020). Targeting the endolysosomal host-SARS-CoV-2 interface by clinically licensed functional inhibitors of acid sphingomyelinase (FIASMA) including the antidepressant fluoxetine. Emerg. Microbes Infect..

[100] Edwards MJ (2020). Sphingosine prevents binding of SARS-CoV-2 spike to its cellular receptor ACE2. J. Biol. Chem..

[101] Hoertel N (2021). Repurposing antidepressants inhibiting the sphingomyelinase acid/ceramide system against COVID-19: current evidence and potential mechanisms. Mol. Psychiatry.

[102] Nguyen L (2022). Sialic acid-containing glycolipids mediate binding and viral entry of SARS-CoV-2. Nat. Chem. Biol..

[103] Fantini J, Di Scala C, Chahinian H, Yahi N (2020). Structural and molecular modelling studies reveal a new mechanism of action of chloroquine and hydroxychloroquine against SARS-CoV-2 infection. Int. J. Antimicrob. Agents.

[104] Yuan, Z., Pavel, M. A., Wang, H. & Hansen, S. B. Hydroxychloroquine: mechanism of action inhibiting SARS-CoV2 entry. Preprint at *bioRxiv*10.1101/2020.08.13.250217 (2020).

[105] Al-Bari MA (2015). Chloroquine analogues in drug discovery: new directions of uses, mechanisms of actions and toxic manifestations from malaria to multifarious diseases. J. Antimicrob. Chemother..

[106] Pastick KA (2020). Review: hydroxychloroquine and chloroquine for treatment of SARS-CoV-2 (COVID-19). Open Forum Infect. Dis..

[107] Das S (2021). The controversial therapeutic journey of chloroquine and hydroxychloroquine in the battle against SARS-CoV-2: A comprehensive review. Med. Drug. Discov..

[108] Zhang Q, Wang Y, Qi C, Shen L, Li J (2020). Clinical trial analysis of 2019-nCoV therapy registered in China. J. Med. Virol..

[109] Gao J, Tian Z, Yang X (2020). Breakthrough: Chloroquine phosphate has shown apparent efficacy in treatment of COVID-19 associated pneumonia in clinical studies. Biosci. Trends.

[110] Brevini T (2022). FXR inhibition may protect from SARS-CoV-2 infection by reducing ACE2. Nature.

[111] Feng T (2022). Glycosylation of viral proteins: Implication in virus-host interaction and virulence. Virulence.

[112] Cheng N (2022). Protein post-translational modification in SARS-CoV-2 and host interaction. Front. Immunol..

[113] Tripathi N, Goel B, Bhardwaj N, Vishwakarma RA, Jain SK (2022). Exploring the potential of chemical inhibitors for targeting post-translational glycosylation of coronavirus (SARS-CoV-2). ACS Omega.

[114] Tortorici MA (2019). Structural basis for human coronavirus attachment to sialic acid receptors. Nat. Struct. Mol. Biol..

[115] Yuan Y (2017). Cryo-EM structures of MERS-CoV and SARS-CoV spike glycoproteins reveal the dynamic receptor binding domains. Nat. Commun..

[116] Wrapp D (2020). Cryo-EM structure of the 2019-nCoV spike in the prefusion conformation. Science.

[117] Benton DJ (2020). Receptor binding and priming of the spike protein of SARS-CoV-2 for membrane fusion. Nature.

[118] Toelzer C (2020). Free fatty acid binding pocket in the locked structure of SARS-CoV-2 spike protein. Science.

[119] Vivar-Sierra A (2021). In silico study of polyunsaturated fatty acids as potential SARS-CoV-2 spike protein closed conformation stabilizers: epidemiological and computational approaches. Molecules.

[120] Sofia FOA (2021). The fatty acid site is coupled to functional motifs in the SARS-CoV-2 spike protein and modulates spike allosteric behaviour. Comput. Struct. Biotechnol. J..

[121] Li X (2022). Protein palmitoylation modification during viral infection and detection methods of palmitoylated proteins. Front. Cell Infect. Microbiol..

[122] Abdulrahman DA, Meng X, Veit M (2021). S-Acylation of proteins of coronavirus and influenza virus: conservation of acylation sites in animal viruses and DHHC acyltransferases in their animal reservoirs. Pathogens.

[123] Yang J (2012). Replication of murine coronavirus requires multiple cysteines in the endodomain of spike protein. Virology.

[124] Bangaru S (2021). Structural analysis of full-length SARS-CoV-2 spike protein from an advanced vaccine candidate. Science.

[125] Li D, Liu Y, Lu Y, Gao S, Zhang L (2022). Palmitoylation of SARS-CoV-2 S protein is critical for S-mediated syncytia formation and virus entry. J. Med. Virol..

[126] Wu Z (2021). Palmitoylation of SARS-CoV-2 S protein is essential for viral infectivity. Signal Transduct. Target. Ther..

[127] Zeng XT, Yu XX, Cheng W (2021). The interactions of ZDHHC5/GOLGA7 with SARS-CoV-2 spike (S) protein and their effects on S protein’s subcellular localization, palmitoylation and pseudovirus entry. Virol. J..

[128] Mesquita FS (2021). S-acylation controls SARS-CoV-2 membrane lipid organization and enhances infectivity. Dev. Cell.

[129] Petit CM (2007). Palmitoylation of the cysteine-rich endodomain of the SARS-coronavirus spike glycoprotein is important for spike-mediated cell fusion. Virology.

[130] McBride CE, Machamer CE (2010). Palmitoylation of SARS-CoV S protein is necessary for partitioning into detergent-resistant membranes and cell-cell fusion but not interaction with M protein. Virology.

[131] Vargas-Rodriguez JR (2022). Sustained hyperglycemia and its relationship with the outcome of hospitalized patients with severe COVID-19: potential role of ACE2 upregulation. J. Pers. Med..

[132] Wysocki J (2006). ACE and ACE2 activity in diabetic mice. Diabetes.

[133] Rao ST, Lau A, So HC (2020). Exploring diseases/traits and blood proteins causally related to expression of ACE2, the putative receptor of SARS-CoV-2: a Mendelian randomization analysis highlights tentative relevance of diabetes-related traits. Diabetes Care.

[134] Romani-Perez M (2015). Activation of the GLP-1 receptor by liraglutide increases ACE2 expression, reversing right ventricle hypertrophy, and improving the production of SP-A and SP-B in the lungs of type 1 diabetes rats. Endocrinology.

[135] Zhang W (2014). Pioglitazone upregulates angiotensin converting enzyme 2 expression in insulin-sensitive tissues in rats with high-fat diet-induced nonalcoholic steatohepatitis. TheScientificWorldJournal.

[136] Wosten-van Asperen RM (2011). Acute respiratory distress syndrome leads to reduced ratio of ACE/ACE2 activities and is prevented by angiotensin-(1-7) or an angiotensin II receptor antagonist. J. Pathol..

[137] Ferrario CM (2005). Effect of angiotensin-converting enzyme inhibition and angiotensin II receptor blockers on cardiac angiotensin-converting enzyme 2. Circulation.

[138] Zou X (2020). Single-cell RNA-seq data analysis on the receptor ACE2 expression reveals the potential risk of different human organs vulnerable to 2019-nCoV infection. Front. Med..

[139] Lukassen S (2020). SARS-CoV-2 receptor ACE2 and TMPRSS2 are primarily expressed in bronchial transient secretory cells. EMBO J..

[140] Garreta E (2022). A diabetic milieu increases ACE2 expression and cellular susceptibility to SARS-CoV-2 infections in human kidney organoids and patient cells. Cell Metab..

[141] Vigerust DJ, Shepherd VL (2007). Virus glycosylation: role in virulence and immune interactions. Trends Microbiol..

[142] Yang TJ (2020). Cryo-EM analysis of a feline coronavirus spike protein reveals a unique structure and camouflaging glycans. Proc. Natl Acad. Sci. USA.

[143] Walls AC (2016). Glycan shield and epitope masking of a coronavirus spike protein observed by cryo-electron microscopy. Nat. Struct. Mol. Biol..

[144] Watanabe Y (2020). Vulnerabilities in coronavirus glycan shields despite extensive glycosylation. Nat. Commun..

[145] Shajahan A, Supekar NT, Gleinich AS, Azadi P (2020). Deducing the N- and O-glycosylation profile of the spike protein of novel coronavirus SARS-CoV-2. Glycobiology.

[146] Sanda M, Morrison L, Goldman R (2021). N- and O-glycosylation of the SARS-CoV-2 spike protein. Anal. Chem..

[147] Zhou D, Tian X, Qi R, Peng C, Zhang W (2021). Identification of 22 N-glycosites on spike glycoprotein of SARS-CoV-2 and accessible surface glycopeptide motifs: implications for vaccination and antibody therapeutics. Glycobiology.

[148] Antonopoulos A (2021). Site-specific characterization of SARS-CoV-2 spike glycoprotein receptor-binding domain. Glycobiology.

[149] Watanabe Y (2020). Site-specific glycan analysis of the SARS-CoV-2 spike. Science.

[150] Li Q (2020). The impact of mutations in SARS-CoV-2 spike on viral infectivity and antigenicity. Cell.

[151] Casalino L (2020). Beyond shielding: the roles of glycans in the SARS-CoV-2 spike protein. ACS Cent. Sci..

[152] Huang HC (2021). Targeting conserved N-glycosylation blocks SARS-CoV-2 variant infection in vitro. EBioMedicine.

[153] Hoffmann M, Kleine-Weber H, Pohlmann S (2020). A multibasic cleavage site in the spike protein of SARS-CoV-2 is essential for infection of human lung cells. Mol. Cell.

[154] Cheng YW (2020). Furin inhibitors block SARS-CoV-2 spike protein cleavage to suppress virus production and cytopathic effects. Cell Rep..

[155] Yang Q (2020). Inhibition of SARS-CoV-2 viral entry upon blocking N- and O-glycan elaboration. Elife.

[156] Zhang L (2021). Furin cleavage of the SARS-CoV-2 spike is modulated by O-glycosylation. Proc. Natl Acad. Sci. USA.

[157] Thaker SK, Ch’ng J, Christofk HR (2019). Viral hijacking of cellular metabolism. BMC Biol..

[158] Twu WI (2021). Contribution of autophagy machinery factors to HCV and SARS-CoV-2 replication organelle formation. Cell Rep..

[159] Koepke L, Hirschenberger M, Hayn M, Kirchhoff F, Sparrer KM (2021). Manipulation of autophagy by SARS-CoV-2 proteins. Autophagy.

[160] Miao G (2021). ORF3a of the COVID-19 virus SARS-CoV-2 blocks HOPS complex-mediated assembly of the SNARE complex required for autolysosome formation. Dev. Cell.

[161] Gassen NC (2019). SKP2 attenuates autophagy through Beclin1-ubiquitination and its inhibition reduces MERS-Coronavirus infection. Nat. Commun..

[162] Gassen NC (2021). SARS-CoV-2-mediated dysregulation of metabolism and autophagy uncovers host-targeting antivirals. Nat. Commun..

[163] Delorme-Axford E, Klionsky DJ (2020). Highlights in the fight against COVID-19: does autophagy play a role in SARS-CoV-2 infection?. Autophagy.

[164] Miller K (2020). Coronavirus interactions with the cellular autophagy machinery. Autophagy.

[165] Malone B, Urakova N, Snijder EJ, Campbell EA (2022). Structures and functions of coronavirus replication-transcription complexes and their relevance for SARS-CoV-2 drug design. Nat. Rev. Mol. Cell. Biol..

[166] Pombo JP, Sanyal S (2018). Perturbation of intracellular cholesterol and fatty acid homeostasis during flavivirus infections. Front. Immunol..

[167] Yan B (2019). Characterization of the lipidomic profile of human coronavirus-infected cells: implications for lipid metabolism remodeling upon coronavirus replication. Viruses.

[168] Heaton NS, Randall G (2011). Multifaceted roles for lipids in viral infection. Trends Microbiol..

[169] Moriel-Carretero M (2020). The hypothetical role of phosphatidic acid in subverting ER membranes during SARS-CoV infection. Traffic.

[170] Tabata K (2021). Convergent use of phosphatidic acid for hepatitis C virus and SARS-CoV-2 replication organelle formation. Nat. Commun..

[171] Yan B (2022). Phosphatidic acid phosphatase 1 impairs SARS-CoV-2 replication by affecting the glycerophospholipid metabolism pathway. Int. J. Biol. Sci..

[172] Xu K, Nagy PD (2015). RNA virus replication depends on enrichment of phosphatidylethanolamine at replication sites in subcellular membranes. Proc. Natl Acad. Sci. USA.

[173] Xu K, Nagy PD (2016). Enrichment of phosphatidylethanolamine in viral replication compartments via co-opting the endosomal Rab5 small GTPase by a positive-strand RNA virus. PLoS Biol..

[174] Belov GA (2015). Less grease, please. phosphatidylethanolamine is the only lipid required for replication of a (+)RNA virus. Viruses.

[175] Huang Q, Lei H, Ding L, Wang Y (2019). Stimulated phospholipid synthesis is key for hepatitis B virus replications. Sci. Rep..

[176] He G (2019). An engineered mutant of a host phospholipid synthesis gene inhibits viral replication without compromising host fitness. J. Biol. Chem..

[177] Williams CG (2021). Inhibitors of VPS34 and fatty-acid metabolism suppress SARS-CoV-2 replication. Cell Rep..

[178] Chu J (2021). Pharmacological inhibition of fatty acid synthesis blocks SARS-CoV-2 replication. Nat. Metab..

[179] Heaton NS (2010). Dengue virus nonstructural protein 3 redistributes fatty acid synthase to sites of viral replication and increases cellular fatty acid synthesis. Proc. Natl Acad. Sci. USA.

[180] Martin-Acebes MA, Jimenez de Oya N, Saiz JC (2019). Lipid metabolism as a source of druggable targets for antiviral discovery against Zika and other flaviviruses. Pharmaceuticals.

[181] Crawford SE, Desselberger U (2016). Lipid droplets form complexes with viroplasms and are crucial for rotavirus replication. Curr. Opin. Virol..

[182] Saka HA, Valdivia R (2012). Emerging roles for lipid droplets in immunity and host-pathogen interactions. Annu. Rev. Cell. Dev. Biol..

[183] Zhang J, Lan Y, Sanyal S (2017). Modulation of lipid droplet metabolism-A potential target for therapeutic intervention in Flaviviridae infections. Front. Microbiol..

[184] Cermelli S, Guo Y, Gross SP, Welte MA (2006). The lipid-droplet proteome reveals that droplets are a protein-storage depot. Curr. Biol..

[185] Farese RV, Walther TC (2009). Lipid droplets finally get a little R-E-S-P-E-C-T. Cell.

[186] Pol A, Gross SP, Parton RG (2014). Review: biogenesis of the multifunctional lipid droplet: lipids, proteins, and sites. J. Cell Biol..

[187] Olzmann JA, Carvalho P (2019). Dynamics and functions of lipid droplets. Nat. Rev. Mol. Cell. Biol..

[188] Pagliari F (2020). ssRNA virus and host lipid rearrangements: is there a role for lipid droplets in SARS-CoV-2 infection?. Front. Mol. Biosci..

[189] Dias SSG (2020). Lipid droplets fuel SARS-CoV-2 replication and production of inflammatory mediators. PLoS Pathog..

[190] Yuan S (2021). SARS-CoV-2 exploits host DGAT and ADRP for efficient replication. Cell Discov..

[191] Fonnesu R (2022). Palmitoylethanolamide (PEA) inhibits SARS-CoV-2 entry by interacting with S protein and ACE-2 receptor. Viruses.

[192] Ricciardi S (2022). The role of NSP6 in the biogenesis of the SARS-CoV-2 replication organelle. Nature.

[193] Moretti F (2018). TMEM41B is a novel regulator of autophagy and lipid mobilization. EMBO Rep..

[194] Li YE (2021). TMEM41B and VMP1 are scramblases and regulate the distribution of cholesterol and phosphatidylserine. J. Cell Biol..

[195] Sun L (2021). Genome-scale CRISPR screen identifies TMEM41B as a multi-function host factor required for coronavirus replication. PLoS Pathog..

[196] Ji M (2022). VMP1 and TMEM41B are essential for DMV formation during beta-coronavirus infection. J. Cell Biol..

[197] Shoemaker CJ (2019). CRISPR screening using an expanded toolkit of autophagy reporters identifies TMEM41B as a novel autophagy factor. PLoS Biol..

[198] Morita K (2018). Genome-wide CRISPR screen identifies TMEM41B as a gene required for autophagosome formation. J. Cell Biol..

[199] Schneider WM (2021). Genome-scale identification of SARS-CoV-2 and Pan-coronavirus host factor networks. Cell.

[200] Trimarco JD (2021). TMEM41B is a host factor required for the replication of diverse coronaviruses including SARS-CoV-2. PLoS pathog..

[201] Kratzel A (2021). A genome-wide CRISPR screen identifies interactors of the autophagy pathway as conserved coronavirus targets. PLoS Biol..

[202] Icard P (2021). The key role of Warburg effect in SARS-CoV-2 replication and associated inflammatory response. Biochimie.

[203] Roy S, Demmer RT (2022). Impaired glucose regulation, SARS-CoV-2 infections and adverse COVID-19 outcomes. Transl. Res..

[204] Montefusco L (2021). Acute and long-term disruption of glycometabolic control after SARS-CoV-2 infection. Nat. Metab..

[205] Sanchez EL, Lagunoff M (2015). Viral activation of cellular metabolism. Virology.

[206] Singh S (2020). AMP-activated protein kinase restricts Zika virus replication in endothelial cells by potentiating innate antiviral responses and inhibiting glycolysis. J. Immunol..

[207] Zhang Y (2021). SARS-CoV-2 hijacks folate and one-carbon metabolism for viral replication. Nat. Commun..

[208] Bojkova D (2020). Proteomics of SARS-CoV-2-infected host cells reveals therapy targets. Nature.

[209] Codo AC (2020). Elevated glucose levels favor SARS-CoV-2 infection and monocyte response through a HIF-1alpha/glycolysis-dependent axis. Cell Metab..

[210] Duan X (2021). An airway organoid-based screen identifies a role for the HIF1alpha-glycolysis axis in SARS-CoV-2 infection. Cell Rep..

[211] Stincone A (2015). The return of metabolism: biochemistry and physiology of the pentose phosphate pathway. Biol. Rev. Camb. Philos. Soc..

[212] Jiang P, Du W, Wu M (2014). Regulation of the pentose phosphate pathway in cancer. Protein Cell.

[213] Santos ESJC (2021). Gene signatures of autopsy lungs from obese patients with COVID-19. Clin. Nutr. Espen..

[214] Bojkova D (2021). Targeting the pentose phosphate pathway for SARS-CoV-2 therapy. Metabolites.

[215] Ducker GS, Rabinowitz JD (2017). One-carbon metabolism in health and disease. Cell Metab..

[216] Hiraoka M, Kagawa Y (2017). Genetic polymorphisms and folate status. Congenit. Anom..

[217] Decroly E, Ferron F, Lescar J, Canard B (2011). Conventional and unconventional mechanisms for capping viral mRNA. Nat. Rev. Microbiol..

[218] Chen Y, Guo D (2016). Molecular mechanisms of coronavirus RNA capping and methylation. Virol. Sin..

[219] Sevajol M, Subissi L, Decroly E, Canard B, Imbert I (2014). Insights into RNA synthesis, capping, and proofreading mechanisms of SARS-coronavirus. Virus Res..

[220] Mandilara G (2021). The role of coronavirus RNA-processing enzymes in innate immune evasion. Life (Basel).

[221] Yan L (2021). Coupling of N7-methyltransferase and 3’-5’ exoribonuclease with SARS-CoV-2 polymerase reveals mechanisms for capping and proofreading. Cell.

[222] Romano M, Ruggiero A, Squeglia F, Maga G, Berisio R (2020). A structural view of SARS-CoV-2 RNA replication machinery: RNA synthesis, proofreading and final capping. Cells.

[223] Mentch SJ, Locasale JW (2016). One-carbon metabolism and epigenetics: understanding the specificity. Ann. N. Y. Acad. Sci..

[224] Bergant V (2022). Attenuation of SARS-CoV-2 replication and associated inflammation by concomitant targeting of viral and host cap 2’-O-ribose methyltransferases. EMBO J..

[225] Jack A (2021). SARS-CoV-2 nucleocapsid protein forms condensates with viral genomic RNA. PLoS Biol..

[226] Luo H (2006). Severe acute respiratory syndrome coronavirus membrane protein interacts with nucleocapsid protein mostly through their carboxyl termini by electrostatic attraction. Int. J. Biochem. Cell Biol..

[227] Neuman BW (2011). A structural analysis of M protein in coronavirus assembly and morphology. J. Struct. Biol..

[228] Thomas S (2020). The structure of the membrane protein of SARS-CoV-2 resembles the sugar transporter SemiSWEET. Pathog. Immun..

[229] Mortola E, Roy P (2004). Efficient assembly and release of SARS coronavirus-like particles by a heterologous expression system. FEBS Lett..

[230] Corse E, Machamer CE (2003). The cytoplasmic tails of infectious bronchitis virus E and M proteins mediate their interaction. Virology.

[231] Baudoux P (1998). Coronavirus pseudoparticles formed with recombinant M and E proteins induce alpha interferon synthesis by leukocytes. J. Virol..

[232] Yuan Z (2022). The E3 ubiquitin ligase RNF5 facilitates SARS-CoV-2 membrane protein-mediated virion release. MBIO.

[233] Cabrera-Garcia D (2021). The envelope protein of SARS-CoV-2 increases intra-Golgi pH and forms a cation channel that is regulated by pH. J. Physiol..

[234] Boson B (2021). The SARS-CoV-2 envelope and membrane proteins modulate maturation and retention of the spike protein, allowing assembly of virus-like particles. J. Biol. Chem..

[235] Eymieux S (2021). Secretory vesicles are the principal means of SARS-CoV-2 Egress. Cells.

[236] Monje-Galvan, V. & Voth, G. A. Molecular interactions of the M and E integral membrane proteins of SARS-CoV-2. *bioRxiv* (2021). 10.1039/d1fd00031d PMC8712422 34543372

[237] Raamsman MJB (2000). Characterization of the coronavirus mouse hepatitis virus strain A59 small membrane protein E. J. Viol..

[238] Veit M (2012). Palmitoylation of virus proteins. Biol. Cell.

[239] Li M, Yang C, Tong S, Weidmann A, Compans RW (2002). Palmitoylation of the murine leukemia virus envelope protein is critical for lipid raft association and surface expression. J. Virol..

[240] Boscarino JA, Logan HL, Lacny JJ, Gallagher TM (2008). Envelope protein palmitoylations are crucial for murine coronavirus assembly. J. Virol..

[241] Resh MD (2016). Fatty acylation of proteins: the long and the short of it. Prog. Lipid Res..

[242] Rana MS, Lee CJ, Banerjee A (2019). The molecular mechanism of DHHC protein acyltransferases. Biochem. Soc. Trans..

[243] Lopez LA, Riffle AJ, Pike SL, Gardner D, Hogue BG (2008). Importance of conserved cysteine residues in the coronavirus envelope protein. J. Virol..

[244] Liao Y, Yuan Q, Torres J, Tam JP, Liu DX (2006). Biochemical and functional characterization of the membrane association and membrane permeabilizing activity of the severe acute respiratory syndrome coronavirus envelope protein. Virology.

[245] Sun S (2021). Computational study on the function of palmitoylation on the envelope protein in SARS-CoV-2. J. Chem. Theory Comput..

[246] Kuzmin A, Orekhov P, Astashkin R, Gordeliy V, Gushchin I (2022). Structure and dynamics of the SARS-CoV-2 envelope protein monomer. Proteins.

[247] Wei Y (2020). Analysis of 2019 novel coronavirus infection and clinical characteristics of outpatients: an epidemiological study from a fever clinic in Wuhan, China. J. Med. Virol..

[248] Lo MW, Kemper C, Woodruff TM (2020). COVID-19: complement, coagulation, and collateral damage. J. Immunol..

[249] Qin C (2020). Dysregulation of immune response in patients with coronavirus 2019 (COVID-19) in Wuhan, China. Clin. Infect. Dis..

[250] Blanco-Melo D (2020). Imbalanced host response to SARS-CoV-2 drives development of COVID-19. Cell.

[251] Wang Y, Wu M, Li Y, Yuen HH, He ML (2022). The effects of SARS-CoV-2 infection on modulating innate immunity and strategies of combating inflammatory response for COVID-19 therapy. J. Biomed. Sci..

[252] Kouwaki T, Nishimura T, Wang G, Oshiumi H (2021). RIG-I-like receptor-mediated recognition of viral genomic RNA of severe acute respiratory syndrome coronavirus-2 and viral escape from the host innate immune responses. Front. Immunol..

[253] Aboudounya MM, Heads RJ (2021). COVID-19 and toll-like receptor 4 (TLR4): SARS-CoV-2 may bind and activate TLR4 to increase ACE2 expression, facilitating entry and causing hyperinflammation. Mediat. Inflamm..

[254] Rodrigues TS (2021). Inflammasomes are activated in response to SARS-CoV-2 infection and are associated with COVID-19 severity in patients. J. Exp. Med..

[255] Toldo S (2021). Inflammasome formation in the lungs of patients with fatal COVID-19. Inflamm. Res..

[256] Wu B (2022). ORAI1 limits SARS-CoV-2 infection by regulating tonic type I IFN signaling. J. Immunol..

[257] Quarleri J, Delpino MV (2021). Type I and III IFN-mediated antiviral actions counteracted by SARS-CoV-2 proteins and host inherited factors. Cytokine Growth Factor Rev..

[258] Lamers MM, Haagmans BL (2022). SARS-CoV-2 pathogenesis. Nat. Rev. Microbiol..

[259] Zheng BJ (2004). Potent inhibition of SARS-associated coronavirus (SCOV) infection and replication by type I interferons (IFN-alpha beta) but not by type II interferon (IFN-gamma). J. Interf. Cytok. Res..

[260] Yongzhi X (2021). COVID-19-associated cytokine storm syndrome and diagnostic principles: an old and new Issue. Emerg. Microbes Infect..

[261] Olbei M (2021). SARS-CoV-2 causes a different cytokine response compared to other cytokine storm-causing respiratory viruses in severely Ill patients. Front. Immunol..

[262] Karki R (2021). Synergism of TNF-alpha and IFN-gamma triggers inflammatory cell death, tissue damage, and mortality in SARS-CoV-2 infection and cytokine shock syndromes. Cell.

[263] Zhou YG (2020). Pathogenic T-cells and inflammatory monocytes incite inflammatory storms in severe COVID-19 patients. Natl Sci. Rev..

[264] Chen G (2020). Clinical and immunological features of severe and moderate coronavirus disease 2019. J. Clin. Invest..

[265] Merad M, Martin JC (2020). Pathological inflammation in patients with COVID-19: a key role for monocytes and macrophages. Nat. Rev. Immunol..

[266] Dolhnikoff M (2020). Pathological evidence of pulmonary thrombotic phenomena in severe COVID-19. J. Thromb. Haemost..

[267] Menter T (2020). Postmortem examination of COVID-19 patients reveals diffuse alveolar damage with severe capillary congestion and variegated findings in lungs and other organs suggesting vascular dysfunction. Histopathology.

[268] Ackermann M (2020). Pulmonary vascular endothelialitis, thrombosis, and angiogenesis in Covid-19. N. Engl. J. Med..

[269] Gu SX (2021). Thrombocytopathy and endotheliopathy: crucial contributors to COVID-19 thromboinflammation. Nat. Rev. Cardiol..

[270] Villar J (2020). Dexamethasone treatment for the acute respiratory distress syndrome: a multicentre, randomised controlled trial. Lancet Respir. Med..

[271] Group RC (2021). Dexamethasone in hospitalized patients with Covid-19. N. Engl. J. Med..

[272] Ehrmann S (2021). Awake prone positioning for COVID-19 acute hypoxaemic respiratory failure: a randomised, controlled, multinational, open-label meta-trial. Lancet Respir. Med..

[273] Zaid Y (2021). Chemokines and eicosanoids fuel the hyperinflammation within the lungs of patients with severe COVID-19. J. Allergy Clin. Immunol..

[274] Kuypers FA (2021). Secretory phospholipase A2 in SARS-CoV-2 infection and multisystem inflammatory syndrome in children (MIS-C). Exp. Biol. Med..

[275] Kott M (2014). Acid Sphingomyelinase Serum Activity Predicts Mortality in Intensive Care Unit Patients after Systemic Inflammation: A Prospective Cohort Study. PLoS ONE.

[276] Ricciotti E, FitzGerald GA (2011). Prostaglandins and inflammation. Arterioscler. Thromb. Vasc. Biol..

[277] Chen, J. S. et al. Cyclooxgenase-2 is induced by SARS-CoV-2 infection but does not affect viral entry or replication. Preprint at *bioRxiv*10.1101/2020.09.24.312769 (2020).

[278] Tan L (2020). Lymphopenia predicts disease severity of COVID-19: a descriptive and predictive study. Signal Transduct. Target. Ther..

[279] Ricke-Hoch M (2021). Impaired immune response mediated by prostaglandin E2 promotes severe COVID-19 disease. PLoS ONE.

[280] Aliabadi F, Ajami M, Pazoki-Toroudi H (2020). Why does COVID-19 pathology have several clinical forms?. BioEssays.

[281] Gupta A, Chander Chiang K (2020). Prostaglandin D2 as a mediator of lymphopenia and a therapeutic target in COVID-19 disease. Med. Hypotheses.

[282] Serhan CN, Chiang N, Van Dyke TE (2008). Resolving inflammation: dual anti-inflammatory and pro-resolution lipid mediators. Nat. Rev. Immunol..

[283] Manne BK (2020). Platelet gene expression and function in patients with COVID-19. Blood.

[284] Manne BK (2018). PDK1 governs thromboxane generation and thrombosis in platelets by regulating activation of Raf1 in the MAPK pathway. J. Thromb. Haemost..

[285] Gelfand EW (2017). Importance of the leukotriene B4-BLT1 and LTB4-BLT2 pathways in asthma. Semin. Immunol..

[286] Hashimoto K (2009). Cysteinyl leukotrienes induce monocyte chemoattractant protein-1 in human monocyte/macrophages via mitogen-activated protein kinase and nuclear factor-kappaB pathways. Int. Arch. Allergy Immunol..

[287] Goodarzi K, Goodarzi M, Tager AM, Luster AD, von Andrian UH (2003). Leukotriene B4 and BLT1 control cytotoxic effector T cell recruitment to inflamed tissues. Nat. Immunol..

[288] Di Gennaro A, Haeggstrom JZ (2012). The leukotrienes: immune-modulating lipid mediators of disease. Adv. Immunol..

[289] Kong M, Zhang H, Cao X, Mao X, Lu Z (2020). Higher level of neutrophil-to-lymphocyte is associated with severe COVID-19. Epidemiol. Infect..

[290] Wilk AJ (2020). A single-cell atlas of the peripheral immune response in patients with severe COVID-19. Nat. Med..

[291] Liao M (2020). Single-cell landscape of bronchoalveolar immune cells in patients with COVID-19. Nat. Med..

[292] Huang L (2004). Leukotriene B4 strongly increases monocyte chemoattractant protein-1 in human monocytes. Arterioscler. Thromb. Vasc. Biol..

[293] Brach MA (1992). Leukotriene B4 transcriptionally activates interleukin-6 expression involving NK-xB and NF-IL6. Eur. J. Immunol..

[294] Liu T (2019). Cysteinyl leukotriene receptor 2 drives lung immunopathology through a platelet and high mobility box 1-dependent mechanism. Mucosal Immunol..

[295] Cummings HE (2013). Cutting edge: Leukotriene C4 activates mouse platelets in plasma exclusively through the type 2 cysteinyl leukotriene receptor. J. Immunol..

[296] Xu X (2020). Imaging and clinical features of patients with 2019 novel coronavirus SARS-CoV-2. Eur. J. Nucl. Med. Mol. Imaging.

[297] Archambault AS (2021). High levels of eicosanoids and docosanoids in the lungs of intubated COVID-19 patients. Faseb J..

[298] Spector AA (2009). Arachidonic acid cytochrome P450 epoxygenase pathway. J. Lipid Res..

[299] Gilroy DW (2016). CYP450-derived oxylipins mediate inflammatory resolution. Proc. Natl Acad. Sci. USA.

[300] Deng Y (2011). Endothelial CYP epoxygenase overexpression and soluble epoxide hydrolase disruption attenuate acute vascular inflammatory responses in mice. FASEB J..

[301] Zhou Y (2017). Soluble epoxide hydrolase inhibitor attenuates lipopolysaccharide-induced acute lung injury and improves survival in mice. Shock.

[302] Arshad H (2019). Decreased plasma phospholipid concentrations and increased acid sphingomyelinase activity are accurate biomarkers for community-acquired pneumonia. J. Transl. Med..

[303] Beckmann N (2017). Regulation of arthritis severity by the acid sphingomyelinase. Cell Physiol. Biochem..

[304] Chung HY (2016). Acid sphingomyelinase promotes endothelial stress response in systemic inflammation and sepsis. Mol. Med..

[305] Pandolfi R (2017). Role of acid sphingomyelinase and IL-6 as mediators of endotoxin-induced pulmonary vascular dysfunction. Thorax.

[306] Berkman SA, Tapson VF (2021). COVID-19 and its implications for thrombosis and anticoagulation. Semin. Respir. Crit. Care Med..

[307] Grover SP, Mackman N (2018). Tissue factor: an essential mediator of hemostasis and trigger of thrombosis. Arterioscler. Thromb. Vasc. Biol..

[308] Wang J (2021). SARS-CoV-2 infection induces the activation of tissue factor-mediated coagulation via activation of acid sphingomyelinase. Blood.

[309] Abusukhun M (2021). Activation of sphingomyelinase-ceramide-pathway in COVID-19 purposes its inhibition for therapeutic strategies. Front. Immunol..

[310] Mostaza JM (2022). Pre-infection HDL-cholesterol levels and mortality among elderly patients infected with SARS-CoV-2. Atherosclerosis.

[311] Hilser JR (2021). Association of serum HDL-cholesterol and apolipoprotein A1 levels with risk of severe SARS-CoV-2 infection. J. Lipid Res..

[312] Masana L (2021). Low HDL and high triglycerides predict COVID-19 severity. Sci. Rep..

[313] Peng F, Lei S, Zhang Q, Zhong Y, Wu S (2022). Triglyceride/high-density lipoprotein cholesterol ratio is associated with the mortality of COVID-19: a retrospective study in China. Int. J. Gen. Med..

[314] Groenen AG, Halmos B, Tall AR, Westerterp M (2021). Cholesterol efflux pathways, inflammation, and atherosclerosis. Crit. Rev. Biochem. Mol. Biol..

[315] Tall AR, Yvan-Charvet L (2015). Cholesterol, inflammation and innate immunity. Nat. Rev. Immunol..

[316] de la Roche M (2018). Trafficking of cholesterol to the ER is required for NLRP3 inflammasome activation. J. Cell Biol..

[317] Kelley N, Jeltema D, Duan Y, He Y (2019). The NLRP3 inflammasome: an overview of mechanisms of activation and regulation. Int. J. Mol. Sci..

[318] Cardoso D, Perucha E (2021). Cholesterol metabolism: a new molecular switch to control inflammation. Clin. Sci..

[319] Radhakrishnan A, Goldstein JL, McDonald JG, Brown MS (2008). Switch-like control of SREBP-2 transport triggered by small changes in ER cholesterol: a delicate balance. Cell Metab..

[320] Guo C (2018). Cholesterol homeostatic regulator SCAP-SREBP2 integrates NLRP3 inflammasome activation and cholesterol biosynthetic signaling in macrophages. Immunity.

[321] Li LC (2013). Cross-talk between TLR4-MyD88-NF-kappaB and SCAP-SREBP2 pathways mediates macrophage foam cell formation. Am. J. Physiol. Heart Circ. Physiol..

[322] Kusnadi A (2019). The cytokine TNF promotes transcription factor SREBP activity and binding to inflammatory genes to activate macrophages and limit tissue repair. Immunity.

[323] Lee W (2020). COVID-19-activated SREBP2 disturbs cholesterol biosynthesis and leads to cytokine storm. Signal Transduct. Target. Ther..

[324] Navab M, Anantharamaiah GM, Fogelman AM (2005). The role of high-density lipoprotein in inflammation. Trends Cardiovasc. Med..

[325] Samadi S (2019). Human T lymphotropic virus type 1 and risk of cardiovascular disease: high-density lipoprotein dysfunction versus serum HDL-C concentrations. Biofactors.

[326] Meilhac O, Tanaka S, Couret D (2020). High-density lipoproteins are bug scavengers. Biomolecules.

[327] Stasi A (2021). Multifaced roles of HDL in sepsis and SARS-CoV-2 infection: renal implications. Int. J. Mol. Sci..

[328] Gao YD (2021). Risk factors for severe and critically ill COVID-19 patients: a review. Allergy.

[329] Chea N (2022). Risk factors for SARS-CoV-2 infection among US healthcare personnel, May-December 2020. Emerg. Infect. Dis..

[330] Wu CT (2021). SARS-CoV-2 infects human pancreatic beta cells and elicits beta cell impairment. Cell Metab..

[331] Shaharuddin SH (2021). Deleterious effects of SARS-CoV-2 infection on human pancreatic cells. Front. Cell Infect. Microbiol..

[332] Liu F (2020). ACE2 expression in pancreas may cause pancreatic damage after SARS-CoV-2 infection. Clin. Gastroenterol. Hepatol..

[333] Khunti K (2021). COVID-19, hyperglycemia, and new-onset diabetes. Diabetes Care.

[334] Donath MY, Dinarello CA, Mandrup-Poulsen T (2019). Targeting innate immune mediators in type 1 and type 2 diabetes. Nat. Rev. Immunol..

[335] Esposito K (2002). Inflammatory cytokine concentrations are acutely increased by hyperglycemia in humans: role of oxidative stress. Circulation.

[336] Hu R, Xia CQ, Butfiloski E, Clare-Salzler M (2018). Effect of high glucose on cytokine production by human peripheral blood immune cells and type I interferon signaling in monocytes: Implications for the role of hyperglycemia in the diabetes inflammatory process and host defense against infection. Clin. Immunol..

[337] Guo W (2020). Diabetes is a risk factor for the progression and prognosis of COVID-19. Diabetes Metab. Res. Rev..

[338] Quinn WJ (2020). Lactate limits T cell proliferation via the NAD(H) redox state. Cell Rep..

[339] Manosalva C (2021). Role of lactate in inflammatory processes: friend or foe. Front. Immunol..

[340] Pucino V (2019). Lactate buildup at the site of chronic inflammation promotes disease by inducing CD4(+) T cell metabolic rewiring. Cell Metab..

[341] Ivashkiv LB (2020). The hypoxia-lactate axis tempers inflammation. Nat. Rev. Immunol..

[342] Carpene G (2022). Blood lactate concentration in COVID-19: a systematic literature review. Clin. Chem. Lab. Med..

[343] Battaglini D, Lopes-Pacheco M, Castro-Faria-Neto HC, Pelosi P, Rocco PRM (2022). Laboratory biomarkers for diagnosis and prognosis in COVID-19. Front. Immunol..

[344] Liang W (2020). Development and validation of a clinical risk score to predict the occurrence of critical illness in hospitalized patients with COVID-19. JAMA Intern. Med..

[345] Zabczyk M (2020). Elevated lactate levels in acute pulmonary embolism are associated with prothrombotic fibrin clot properties: contribution of NETs formation. J. Clin. Med..

[346] Lee S (2021). Virus-induced senescence is a driver and therapeutic target in COVID-19. Nature.

[347] Colegio OR (2014). Functional polarization of tumour-associated macrophages by tumour-derived lactic acid. Nature.

[348] Kozlov AM, Lone A, Betts DH, Cumming RC (2020). Lactate preconditioning promotes a HIF-1alpha-mediated metabolic shift from OXPHOS to glycolysis in normal human diploid fibroblasts. Sci. Rep..

[349] Zhang W (2019). Lactate is a natural suppressor of RLR signaling by targeting MAVS. Cell.

[350] Gevers D (2014). The treatment-naive microbiome in new-onset Crohn’s disease. Cell Host Microbe.

[351] Yamamoto EA, Jorgensen TN (2019). Relationships between vitamin D, gut microbiome, and systemic autoimmunity. Front. Immunol..

[352] Schirmer M (2016). Linking the human gut microbiome to inflammatory cytokine production capacity. Cell.

[353] Zuo T (2020). Alterations in gut microbiota of patients with COVID-19 during time of hospitalization. Gastroenterology.

[354] Hazan S (2022). Lost microbes of COVID-19: Bifidobacterium, Faecalibacterium depletion and decreased microbiome diversity associated with SARS-CoV-2 infection severity. BMJ Open Gastroenterol..

[355] Yeoh YK (2021). Gut microbiota composition reflects disease severity and dysfunctional immune responses in patients with COVID-19. Gut.

[356] Sykes DL (2021). Post-COVID-19 symptom burden: what is long-COVID and how should we manage it?. Lung.

[357] Lechner-Scott J, Levy M, Hawkes C, Yeh A, Giovannoni G (2021). Long COVID or post COVID-19 syndrome. Mult. Scler. Relat. Disord..

[358] Ferreira-Junior AS (2022). Detection of intestinal dysbiosis in post-COVID-19 patients one to eight months after acute disease resolution. Int. J. Environ. Res. Public Health.

[359] Liu Q (2022). Gut microbiota dynamics in a prospective cohort of patients with post-acute COVID-19 syndrome. Gut.

[360] Rishi P (2020). Diet, Gut Microbiota and COVID-19. Indian J. Microbiol..

[361] Calder PC (2020). Nutrition, immunity and COVID-19. BMJ Nutr. Prev. Health.

[362] Jabczyk M, Nowak J, Hudzik B, Zubelewicz-Szkodzinska B (2021). Diet, probiotics and their impact on the gut microbiota during the COVID-19 pandemic. Nutrients.

[363] Alberca GGF, Alberca RW (2021). Nutrition and the microbiota post-COVID-19. Saudi. J. Gastroenterol..

[364] Pettigrew MM, Tanner W, Harris AD (2021). The lung microbiome and pneumonia. J. Infect. Dis..

[365] Dumas A, Bernard L, Poquet Y, Lugo-Villarino G, Neyrolles O (2018). The role of the lung microbiota and the gut-lung axis in respiratory infectious diseases. Cell Microbiol..

[366] Ceban F (2022). Fatigue and cognitive impairment in Post-COVID-19 Syndrome: a systematic review and meta-analysis. Brain Behav. Immun..

[367] Holdsworth DA (2022). Comprehensive clinical assessment identifies specific neurocognitive deficits in working-age patients with long-COVID. PLoS ONE.

[368] Woo MS (2020). Frequent neurocognitive deficits after recovery from mild COVID-19. Brain Commun..

[369] Raman B, Bluemke DA, Luscher TF, Neubauer S (2022). Long COVID: post-acute sequelae of COVID-19 with a cardiovascular focus. Eur. Heart J..

[370] Mehandru S, Merad M (2022). Pathological sequelae of long-haul COVID. Nat. Immunol..

[371] Davis HE, McCorkell L, Vogel JM, Topol EJ (2023). Long COVID: major findings, mechanisms and recommendations. Nat. Rev. Microbiol..

[372] Raveendran AV, Jayadevan R, Sashidharan S (2021). Long COVID: an overview. Diabetes Metab. Syndr..

[373] Castanares-Zapatero D (2022). Pathophysiology and mechanism of long COVID: a comprehensive review. Ann. Med..

[374] Paul BD, Lemle MD, Komaroff AL, Snyder SH (2021). Redox imbalance links COVID-19 and myalgic encephalomyelitis/chronic fatigue syndrome. Proc. Natl Acad. Sci. USA.

[375] Wong TL, Weitzer DJ (2021). Long COVID and Myalgic Encephalomyelitis/Chronic Fatigue Syndrome (ME/CFS)-A Systemic Review and Comparison of Clinical Presentation and Symptomatology. Med. (Kaunas.).

[376] Castro-Marrero J (2021). Effect of Dietary Coenzyme Q10 Plus NADH Supplementation on Fatigue Perception and Health-Related Quality of Life in Individuals with Myalgic Encephalomyelitis/Chronic Fatigue Syndrome: A Prospective, Randomized, Double-Blind, Placebo-Controlled Trial. Nutrients.

[377] Weinstock LB (2021). Mast cell activation symptoms are prevalent in Long-COVID. Int. J. Infect. Dis..

[378] Wechsler JB, Butuci M, Wong A, Kamboj AP, Youngblood BA (2022). Mast cell activation is associated with post-acute COVID-19 syndrome. Allergy.

[379] Pinto MD (2022). Antihistamines for postacute sequelae of SARS-CoV-2 infection. J. Nurse Pract..

[380] Glynne P, Tahmasebi N, Gant V, Gupta R (2022). Long COVID following mild SARS-CoV-2 infection: characteristic T cell alterations and response to antihistamines. J. Investig. Med..

[381] Bardelcikova A, Mirossay A, Soltys J, Mojzis J (2022). Therapeutic and prophylactic effect of flavonoids in post-COVID-19 therapy. Phytother. Res..

[382] Wright J, Astill SL, Sivan M (2022). The relationship between physical activity and long COVID: a cross-sectional study. Int. J. Environ. Res. Public. Health.

[383] Wang C (2022). Long COVID: the nature of thrombotic sequelae determines the necessity of early anticoagulation. Front. Cell Infect. Microbiol..

[384] Comelli A (2022). Patient-Reported Symptoms and Sequelae 12 Months After COVID-19 in Hospitalized Adults: A Multicenter Long-Term Follow-Up Study. Front. Med..

[385] Law MR, Wald NJ, Rudnicka AR (2003). Quantifying effect of statins on low density lipoprotein cholesterol, ischaemic heart disease, and stroke: systematic review and meta-analysis. Br. Med. J..

[386] Jiang W, Hu JW, He XR, Jin WL, He XY (2021). Statins: a repurposed drug to fight cancer. J. Exp. Clin. Cancer Res..

[387] Fan J (2020). Letter to the Editor: Low-density lipoprotein is a potential predictor of poor prognosis in patients with coronavirus disease 2019. Metabolism.

[388] Oesterle A, Laufs U, Liao JK (2017). Pleiotropic effects of statins on the cardiovascular system. Circ. Res..

[389] McAuley DF (2014). Simvastatin in the acute respiratory distress syndrome. N. Engl. J. Med..

[390] Truwit JD (2014). Rosuvastatin for sepsis-associated acute respiratory distress syndrome. N. Engl. J. Med..

[391] Parihar SP, Guler R, Brombacher F (2019). Statins: a viable candidate for host-directed therapy against infectious diseases. Nat. Rev. Immunol..

[392] Feng Y (2018). Efficacy of statin therapy in patients with acute respiratory distress syndrome_acute lung injury. Eur. Rev. Med. Pharmacol. Sci..

[393] Zhang XJ (2020). In-hospital use of statins is associated with a reduced risk of mortality among individuals with COVID-19. Cell Mateb.

[394] Rodriguez-Nava G (2020). Atorvastatin associated with decreased hazard for death in COVID-19 patients admitted to an ICU: a retrospective cohort study. Crit. Care.

[395] Yetmar ZA (2021). Prior statin use and risk of mortality and severe disease from coronavirus disease 2019: a systematic review and meta-analysis. Open Forum Infect. Dis..

[396] Hariyanto TI, Kurniawan A (2020). Statin therapy did not improve the in-hospital outcome of coronavirus disease 2019 (COVID-19) infection. Int. J. Diabetes Metab. Syndr..

[397] Tikoo K (2015). Tissue specific up regulation of ACE2 in rabbit model of atherosclerosis by atorvastatin: role of epigenetic histone modifications. Biochem. Pharm..

[398] Kornhuber J, Tripal P, Gulbins E, Muehlbacher M (2013). Functional inhibitors of acid sphingomyelinase (FIASMAs). Handb. Exp. Pharm..

[399] Zhemkov V (2021). The role of sigma 1 receptor in organization of endoplasmic reticulum signaling microdomains. Elife.

[400] Vela JM (2020). Repurposing Sigma-1 Receptor Ligands for COVID-19 Therapy?. Front. Pharmacol..

[401] Ishima T, Fujita Y, Hashimoto K (2014). Interaction of new antidepressants with sigma-1 receptor chaperones and their potentiation of neurite outgrowth in PC12 cells. Eur. J. Pharmacol..

[402] Hoertel N (2021). Association Between FIASMAs and Reduced Risk of Intubation or Death in Individuals Hospitalized for Severe COVID-19: An Observational Multicenter Study. Clin. Pharmacol. Ther..

[403] Darquennes G, Le Corre P, Le Moine O, Loas G (2021). Association between Functional Inhibitors of Acid Sphingomyelinase (FIASMAs) and Reduced Risk of Death in COVID-19 Patients: A Retrospective Cohort Study. Pharmaceuticals.

[404] Lenze EJ (2020). Fluvoxamine vs Placebo and Clinical Deterioration in Outpatients With Symptomatic COVID-19: A Randomized Clinical Trial. JAMA.

[405] Reis G (2022). Effect of early treatment with fluvoxamine on risk of emergency care and hospitalisation among patients with COVID-19: the TOGETHER randomised, platform clinical trial. Lancet Glob. Health.

[406] Freites Nunez DD (2020). Risk factors for hospital admissions related to COVID-19 in patients with autoimmune inflammatory rheumatic diseases. Ann. Rheum. Dis..

[407] Rentsch, C. T. et al. Covid-19 testing, hospital admission, and intensive care among 2,026,227 United States Veterans aged 54-75 years. Preprint at *medRxiv*10.1101/2020.04.09.20059964 (2020).

[408] de Bruin N (2022). Ibuprofen, flurbiprofen, etoricoxib or paracetamol do not influence ACE2 expression and activity in vitro or in mice and do not exacerbate in-vitro SARS-CoV-2 infection. Int. J. Mol. Sci..

[409] Chen JS (2021). Non-steroidal anti-inflammatory drugs dampen the cytokine and antibody response to SARS-CoV-2 infection. J. Virol..

[410] Sodhi M, Etminan M (2020). Safety of ibuprofen in patients with COVID-19: causal or confounded?. Chest.

[411] Moore N, Bosco-Levy P, Thurin N, Blin P, Droz-Perroteau C (2021). NSAIDs and COVID-19: a systematic review and meta-analysis. Drug Saf..

[412] Schmidt M (2011). Non-steroidal anti-inflammatory drug use and risk of venous thromboembolism. J. Thromb. Haemost..

[413] Rabausch K (2005). Regulation of thrombomodulin expression in human vascular smooth muscle cells by COX-2-derived prostaglandins. Circ. Res..

[414] FitzGerald GA (2020). Misguided drug advice for COVID-19. Science.

[415] Maeba S (2005). Effect of montelukast on nuclear factor kB activation and proinflammatory molecules. Ann. Allergy Asthma Immunol..

[416] Tahan F, Jazrawi E, Moodley T, Rovati GE, Adcock IM (2008). Montelukast inhibits tumour necrosis factor-alpha-mediated interleukin-8 expression through inhibition of nuclear factor-kappaB p65-associated histone acetyltransferase activity. Clin. Exp. Allergy..

[417] Camera M, Canzano P, Brambilla M, Rovati GE (2022). Montelukast inhibits platelet activation induced by plasma from COVID-19 patients. Front. Pharmacol..

[418] Luedemann M (2022). Montelukast is a dual-purpose inhibitor of SARS-CoV-2 infection and virus-induced IL-6 expression identified by structure-based drug repurposing. Comput. Struct. Biotechnol. J..

[419] Thoms M (2020). Structural basis for translational shutdown and immune evasion by the Nsp1. Science.

[420] Afsar M (2022). Drug targeting Nsp1-ribosomal complex shows antiviral activity against SARS-CoV-2. Elife.

[421] Khan AR (2022). Montelukast in hospitalized patients diagnosed with COVID-19. J. Asthma.

[422] Kerget B, Kerget F, Aydin M, Karasahin O (2022). Effect of montelukast therapy on clinical course, pulmonary function, and mortality in patients with COVID-19. J. Med. Virol..

[423] Serhan CN (2014). Pro-resolving lipid mediators are leads for resolution physiology. Nature.

[424] Serhan CN (2017). Treating inflammation and infection in the 21st century: new hints from decoding resolution mediators and mechanisms. Faseb. J..

[425] Regidor PA, Santos FG, Rizo JM, Egea FM (2020). Pro resolving inflammatory effects of the lipid mediators of omega 3 fatty acids and its implication in SARS COVID-19. Med. Hypotheses.

[426] Ramirez-Santana M (2022). Inverse association between omega-3 index and severity of COVID-19: a case-control study. Int. J. Environ. Res. Public Health.

[427] Doaei S (2021). The effect of omega-3 fatty acid supplementation on clinical and biochemical parameters of critically ill patients with COVID-19: a randomized clinical trial. J. Transl. Med..

[428] Weill P, Plissonneau C, Legrand P, Rioux V, Thibault R (2020). May omega-3 fatty acid dietary supplementation help reduce severe complications in Covid-19 patients?. Biochimie.

[429] Mesri EA, Lampidis TJ (2021). 2-Deoxy-d-glucose exploits increased glucose metabolism in cancer and viral-infected cells: relevance to its use in India against SARS-CoV-2. IUBMB Life.

[430] Sasaki K (2021). Nanoparticle-mediated delivery of 2-deoxy-d-glucose induces antitumor immunity and cytotoxicity in liver tumors in mice. Cell Mol. Gastroenterol. Hepatol..

[431] Rho JM, Shao LR, Stafstrom CE (2019). 2-Deoxyglucose and beta-hydroxybutyrate: metabolic agents for seizure control. Front. Cell Neurosci..

[432] Bhatt AN (2021). Glycolytic inhibitor 2-deoxy-D-glucose attenuates SARS-CoV-2 multiplication in host cells. Life Sci..

[433] Dwarakanath BS, Jain V (2009). Targeting glucose metabolism with 2-deoxy-D-glucose for improving cancer therapy. Future Oncol..

[434] Voss M (2018). Rescue of 2-deoxyglucose side effects by ketogenic diet. Int. J. Mol. Sci..

[435] Bhatt AN (2022). 2-Deoxy-D-glucose as an adjunct to standard of care in the medical management of COVID-19: a proof-of-concept and dose-ranging randomised phase II clinical trial. BMC Infect. Dis..

[436] Bai B, Chen H (2021). Metformin: a novel weapon against inflammation. Front. Pharmacol..

[437] Yi Y (2017). Metformin promotes AMP-activated protein kinase-independent suppression of delta Np63 alpha protein expression and inhibits cancer cell viability. J. Biol. Chem..

[438] Yi Y, Zhang W, Yi J, Xiao ZX (2019). Role of p53 family proteins in metformin anti-cancer activities. J. Cancer.

[439] Wang C, Chen B, Feng Q, Nie C, Li T (2020). Clinical perspectives and concerns of metformin as an anti-aging drug. Aging Med..

[440] Khunti K (2021). Prescription of glucose-lowering therapies and risk of COVID-19 mortality in people with type 2 diabetes: a nationwide observational study in England. Lancet Diabetes Endocrinol..

[441] Lalau JD (2021). Metformin use is associated with a reduced risk of mortality in patients with diabetes hospitalised for COVID-19. Diabetes Metab..

[442] Luo P (2020). Metformin treatment was associated with decreased mortality in COVID-19 patients with diabetes in a retrospective analysis. Am. J. Trop. Med. Hyg..

[443] Chen Y (2020). Clinical characteristics and outcomes of patients with diabetes and COVID-19 in association with glucose-lowering medication. Diabetes Care.

[444] Afshari K (2018). Anti-inflammatory effects of Metformin improve the neuropathic pain and locomotor activity in spinal cord injured rats: introduction of an alternative therapy. Spinal Cord..

[445] Jadhav S (2006). Effects of metformin on microvascular function and exercise tolerance in women with angina and normal coronary arteries: a randomized, double-blind, placebo-controlled study. J. Am. Coll. Cardiol..

[446] Ursini F (2018). Metformin and autoimmunity: a “new deal” of an old drug. Front. Immunol..

[447] Rangarajan S (2018). Metformin reverses established lung fibrosis in a bleomycin model. Nat. Med..

[448] Rena G, Hardie DG, Pearson ER (2017). The mechanisms of action of metformin. Diabetologia.

[449] Choi KS, Aizaki H, Lai MM (2005). Murine coronavirus requires lipid rafts for virus entry and cell-cell fusion but not for virus release. J. Virol..

[450] Guo H (2017). The important role of lipid raft-mediated attachment in the infection of cultured cells by coronavirus infectious bronchitis virus beaudette strain. PLoS ONE.

[451] Wei X, She G, Wu T, Xue C, Cao Y (2020). PEDV enters cells through clathrin-, caveolae-, and lipid raft-mediated endocytosis and traffics via the endo-/lysosome pathway. Vet. Res..

[452] Sviridov D, Miller YI, Ballout RA, Remaley AT, Bukrinsky M (2020). Targeting lipid rafts-a potential therapy for COVID-19. Front. Immunol..

[453] Zhang R, Wang Q, Yang J (2022). Potential of sphingosine-1-phosphate in preventing SARS-CoV-2 infection by stabilizing and protecting endothelial cells. Medicine.

[454] Maceyka M, Spiegel S (2014). Sphingolipid metabolites in inflammatory disease. Nature.

[455] Shulla A, Gallagher T (2009). Role of spike protein endodomains in regulating coronavirus entry. J. Biol. Chem..

[456] Zhang JT (2016). Positive-strand RNA viruses stimulate host phosphatidylcholine synthesis at viral replication sites. Proc. Natl Acad. Sci. USA.

[457] Lee JY (2019). Spatiotemporal coupling of the hepatitis C virus replication cycle by creating a lipid droplet- proximal membranous replication compartment. Cell Rep..

[458] Bang BR (2019). Regulation of hepatitis C virus infection by cellular retinoic acid binding proteins through the modulation of lipid droplet abundance. J. Virol..

[459] Criglar JM, Estes MK, Crawford SE (2022). Rotavirus-induced lipid droplet biogenesis is critical for virus replication. Front. Physiol..

[460] Martins AS, Martins IC, Santos NC (2018). Methods for lipid droplet biophysical characterization in flaviviridae infections. Front. Microbiol..

[461] Zandi M (2022). Interplay between cellular metabolism and DNA viruses. J. Med. Virol..

[462] Martinez-Ramirez I (2018). Regulation of cellular metabolism by high-risk human papillomaviruses. Int. J. Mol. Sci..

[463] Park GJ (2022). The mechanism of RNA capping by SARS-CoV-2. Nature.

[464] Walker AP (2021). The SARS-CoV-2 RNA polymerase is a viral RNA capping enzyme. Nucleic Acids Res..

[465] Bracquemond D, Muriaux D (2021). Betacoronavirus assembly: clues and perspectives for elucidating SARS-CoV-2 particle formation and egress. mBio.

[466] Wang X, Melino G, Shi Y (2021). Actively or passively deacidified lysosomes push beta-coronavirus egress. Cell Death Dis..

[467] Henne M (2019). And three’s a party: lysosomes, lipid droplets, and the ER in lipid trafficking and cell homeostasis. Curr. Opin. Cell Biol..

[468] Jaishy B, Abel ED (2016). Lipids, lysosomes, and autophagy. J. Lipid Res..

[469] Xu L, Yang CS, Liu Y, Zhang X (2022). Effective regulation of gut microbiota with probiotics and prebiotics may prevent or alleviate COVID-19 through the gut-lung axis. Front. Pharmacol..

[470] de Oliveira GLV, Oliveira CNS, Pinzan CF, de Salis LVV, Cardoso CRB (2021). Microbiota modulation of the gut-lung axis in COVID-19. Front. Immunol..

[471] Zhang M (2021). COVID-19: gastrointestinal symptoms from the view of gut-lung axis. Eur. J. Gastroenterol. Hepatol..

